# Antiviral efficacy of favipiravir against Ebola virus: A translational study in cynomolgus macaques

**DOI:** 10.1371/journal.pmed.1002535

**Published:** 2018-03-27

**Authors:** Jérémie Guedj, Géraldine Piorkowski, Frédéric Jacquot, Vincent Madelain, Thi Huyen Tram Nguyen, Anne Rodallec, Stephan Gunther, Caroline Carbonnelle, France Mentré, Hervé Raoul, Xavier de Lamballerie

**Affiliations:** 1 IAME, UMR 1137, INSERM, Université Paris Diderot, Sorbonne Paris Cité, Paris, France; 2 UMR Émergence des Pathologies Virales, Aix-Marseille University, IRD 190, Inserm 1207, École des Hautes Études en Santé Publique, Marseille, France; 3 Laboratoire P4 Inserm–Jean Mérieux, US003 Inserm, Lyon, France; 4 SMARTc Unit, U911 Cro2, Aix-Marseille University, Marseille, France; 5 Bernhard Nocht Institute for Tropical Medicine, Hamburg, Germany; Groote Schuur Hospital, SOUTH AFRICA

## Abstract

**Background:**

Despite repeated outbreaks, in particular the devastating 2014–2016 epidemic, there is no effective treatment validated for patients with Ebola virus disease (EVD). Among the drug candidates is the broad-spectrum polymerase inhibitor favipiravir, which showed a good tolerance profile in patients with EVD (JIKI trial) but did not demonstrate a strong antiviral efficacy. In order to gain new insights into the antiviral efficacy of favipiravir and improve preparedness and public health management of future outbreaks, we assess the efficacy achieved by ascending doses of favipiravir in Ebola-virus-infected nonhuman primates (NHPs).

**Methods and findings:**

A total of 26 animals (*Macaca fascicularis*) were challenged intramuscularly at day 0 with 1,000 focus-forming units of Ebola virus Gabon 2001 strain and followed for 21 days (study termination). This included 13 animals left untreated and 13 treated with doses of 100, 150, and 180 mg/kg (*N =* 3, 5, and 5, respectively) favipiravir administered intravenously twice a day for 14 days, starting 2 days before infection. All animals left untreated or treated with 100 mg/kg died within 10 days post-infection, while animals receiving 150 and 180 mg/kg had extended survival (*P <* 0.001 and 0.001, respectively, compared to untreated animals), leading to a survival rate of 40% (2/5) and 60% (3/5), respectively, at day 21. Favipiravir inhibited viral replication (molecular and infectious viral loads) in a drug-concentration-dependent manner (*P* values < 0.001), and genomic deep sequencing analyses showed an increase in virus mutagenesis over time. These results allowed us to identify that plasma trough favipiravir concentrations greater than 70–80 μg/ml were associated with reduced viral loads, lower virus infectivity, and extended survival. These levels are higher than those found in the JIKI trial, where patients had median trough drug concentrations equal to 46 and 26 μg/ml at day 2 and day 4 post-treatment, respectively, and suggest that the dosing regimen in the JIKI trial was suboptimal. The environment of a biosafety level 4 laboratory introduces a number of limitations, in particular the difficulty of conducting blind studies and performing detailed pharmacological assessments. Further, the extrapolation of the results to patients with EVD is limited by the fact that the model is fully lethal and that treatment initiation in patients with EVD is most often initiated several days after infection, when symptoms and high levels of viral replication are already present.

**Conclusions:**

Our results suggest that favipiravir may be an effective antiviral drug against Ebola virus that relies on RNA chain termination and possibly error catastrophe. These results, together with previous data collected on tolerance and pharmacokinetics in both NHPs and humans, support a potential role for high doses of favipiravir for future human interventions.

## Author summary

**Why was this study done?:**

- In spite of repeated outbreaks, including the devastating 2014–2016 West Africa epidemic, there are no approved antiviral therapeutics against Ebola virus (EBOV).
- In 2014, the European consortium Reaction! was established to assess the anti-EBOV efficacy of favipiravir, a broad-spectrum RNA virus polymerase inhibitor approved for treatment of influenza in Japan.
- The consortium implemented the JIKI clinical trial, which showed that the drug was well tolerated but did not achieve a high antiviral efficacy.
- We here further aimed to determine whether the antiviral efficacy of favipiravir could be improved with higher doses, using a model of EBOV infection in nonhuman primates.

**What did the researchers do and find?:**

- Ascending doses of favipiravir were administered to cynomolgus macaques intravenously twice a day for 14 days, starting 2 days before infection with EBOV.
- Favipiravir inhibited EBOV viral production in a concentration-dependent manner, and doses of 150 and 180 mg/kg led to a survival rate of approximately 50%, allowing us to identify target drug concentrations.
- In addition, EBOV genomic deep sequencing showed an increase in virus mutagenesis over time with favipiravir treatment.

**What do these findings mean?:**

- Our results suggest that favipiravir may be an effective antiviral drug against EBOV that relies on RNA chain termination and possibly error catastrophe.
- These results, together with previous data collected on tolerance and pharmacokinetics in both nonhuman primates and humans, support a potential role for high doses of favipiravir for future human interventions.

## Introduction

The 2014–2016 Ebola virus disease (EVD) outbreak in West Africa has been the deadliest occurrence of the disease since its discovery in 1976. Between January 2014 and June 2016, the World Health Organization (WHO) reported 28,616 EVD cases, of which 11,310 were fatal [1]. There is no validated therapeutic protocol against EVD to date, and none of the therapeutic trials in humans performed during the epidemic, using small molecules [2,3], monoclonal antibodies [4], siRNA [5], or convalescent plasma [6], could demonstrate a statistically significant reduction of mortality in EVD. It is therefore of utmost importance to identify and validate effective treatments for EVD.

In September 2014, a European research consortium (Reaction!) was set up to assess the antiviral efficacy of favipiravir against Ebola virus (EBOV). Favipiravir (T-705) is an RNA polymerase inhibitor approved in Japan for the treatment of non-complicated influenza infections and currently under clinical development in the US. It was one of the prominent candidates identified by WHO for testing in patients with EVD [7] and the only molecule to meet the 3 following criteria: (i) documented antiviral activity against EBOV in vitro and in a laboratory mouse model [8,9], (ii) favorable safety profile when administered to more than 2,000 healthy volunteers or influenza patients worldwide [10], and (iii) immediate availability in large quantities.

The Reaction! consortium implemented a clinical trial to assess the tolerance and the efficacy of favipiravir in Guinean patients with EVD and, in parallel, developed an EBOV infection model in cynomolgus macaques (*Macaca fascicularis*) to assess in detail the efficacy and the mechanism of action of favipiravir [11]. The historically controlled, single-arm proof-of-concept JIKI clinical trial was the largest clinical trial performed during the 2014–2016 epidemic, with more than 100 patients enrolled between November 2014 and April 2015 [2]. Favipiravir had a good tolerance profile but did not demonstrate a strong antiviral efficacy, presumably due to a suboptimal dosing regimen that led to plasma drug concentrations below the drug half maximal effective concentration (EC_50_) [12]. This suggested that higher therapeutic doses of favipiravir would be needed, emphasizing the requirement to determine in vivo the relevant drug concentrations. Accordingly pharmacokinetic (PK) and tolerance studies were performed in uninfected nonhuman primates (NHPs), which allowed us to identify doses of favipiravir having the potential to strongly inhibit EBOV replication in vivo [13].

Here we present the results of studies performed in NHPs infected with EBOV in which favipiravir was used at ascending doses. The virology and survival results are complemented by genomic and PK analysis to provide novel insights into the antiviral mechanism of action of favipiravir against EBOV and to support the evaluation of high doses of favipiravir in future human therapeutic interventions in EVD.

## Methods

### Chronology of the experiments

We present the results of 3 successive experiments conducted in NHPs to evaluate ascending doses of favipiravir of 100, 150, and 180 mg/kg twice a day (BID) that took place in January 2015 (study 1, *N =* 3 treated, *N =* 3 untreated), April 2016 (study 2, *N =* 5 treated, *N =* 5 untreated), and September 2016 (study 3, *N =* 5 treated, *N =* 5 untreated), respectively.

All experiments were performed in the Inserm–Jean Mérieux biosafety level 4 (BSL4) laboratory in Lyon, where animals were housed and monitored in accordance with the guidelines of European Directive 2010/63/EU and procedures established for use of animals in BSL4 facilities. Protocols and experiments received ethical authorization, number 2017APAFIS#6097–2016062713281115 and CECCAPP C2EA15, registered with the French Ministry of Higher Education, Research and Innovation. Excerpts of the protocol submitted for study funding are given in S4 Text.

### Choosing the doses of favipiravir

The first dose tested was 100 mg/kg BID favipiravir, based on PK studies conducted by the drug manufacturer in Chinese-origin cynomolgus macaques, giving average concentrations of 102–158 μg/ml (comparable to the 83 μg/ml targeted in the JIKI trial [13,14]) while maintaining minimal concentrations above the protein-bounded adjusted EC_50_ (21 μg/ml [8]). When the experiment was performed (study 1), a decrease in viral load levels at days 5 and 7 was observed compared to untreated animals (S1 Text), but there was no benefit in terms of survival. In addition, the trough drug concentrations were lower than expected, with median values of 11.5 (min–max 0.4–51.8) and 4.5 (0.4–9.2) μg/ml at days 5 and 7, respectively (S2 Text). This result is consistent with a PK study that was conducted simultaneously with Mauritian uninfected cynomolgus macaques [13], suggesting that drug pharmacokinetics could be different in Chinese- and Mauritian-origin animals, with the latter requiring higher doses of favipiravir. An additional study in uninfected NHPs showed that 150 and 180 mg/kg BID (with a 1-day loading dose of 250 mg/kg) achieved high concentrations of favipiravir in these animals and were well tolerated [13]. By mathematically analyzing data generated in study 1 (*N =* 12), in a study in infected untreated animals [11] (*N =* 15), and in uninfected treated animals [13] (*N =* 30), we predicted that 150 and 180 mg/kg BID could lead to median viral load at day 7 equal to 5.89 (95% prediction interval 5.59–6.29) and 4.40 (3.06–5.19) log_10_ RNA copies/ml, respectively (S1 Text). These predictions, in line with the values found in NHPs successfully treated with ZMapp [15], where all treated animals had a viremia between 3 and 6 log_10_ RNA copies/ml at day 7, suggested that maintenance doses of 150 and 180 mg/kg BID had the potential to lead to extended survival time, giving us the rationale to conduct studies 2 and 3.

### Experimental design

#### Animal care

The experiments were performed using the same procedure and settings as previously described in untreated animals [11]. In brief, female cynomolgus macaques (*M*. *fascicularis*) were obtained from a Mauritian colony free of herpes B virus, tuberculosis, simian T cell leukemia virus, and simian type D retrovirus. Median age was 3 years, and weight ranged from 2.8 to 4.0 kg. Prior to each experiment, the animals were quarantined by Silabe ADUEIS (Strasbourg, France) and then were acclimatized to BSL4 conditions during 7 days with access to food (100 g/day), water (750 ml/day), fruit, and sweets. Animals were housed in individual primate cages that enabled social interactions, under controlled conditions of humidity, temperature, and light (12 hours light and 12 hours dark). Animals were fed with pellets (OWM1 Dietex banana, Dietex International) and with fresh and dry fruits. Enrichment was provided with surprise bags containing sweets and toys. Cages were placed in a ventilated safety cabinet equipped with HEPA filter. Animals were observed by a video surveillance system at least once a day to monitor for disease signs and to assess posture and activity. A clinical disease score was determined daily from day 5 post-infection that included temperature, increase or decrease in food and water intake, weight loss, dehydration, hemorrhage, and rash. A score ≥ 15 was the criterion for alleviating unnecessary suffering of infected animals, and clinical euthanasia of clinically moribund animals was performed by intracardiac administration of 5 ml of pentobarbital under anesthesia. Necropsy was performed for all animals at the end of the follow-up. Study personnel responsible for animal health (including euthanasia) and treatment administration were not blinded to treatment group. Animals did not receive any supportive care treatment.

#### Infection

At day 0, all primates were anesthetized via intramuscular injection using Zoletil (tiletamine/zolazepam, 3 mg/kg) following a protocol described elsewhere [13] and infected by intramuscular injection in the right leg quadriceps with a titrated supernatant fluid containing 1,000 focus-forming units (ffu) of Ebola virus Gabon 2001 strain (a central African strain of EBOV).

#### Treatment

For each experiment, animals were balanced for body weight and age in order to have age and weight uniformity in treated and untreated animals. Following the protocol described previously in treated uninfected animals [13], animals were anesthetized 45 minutes before each drug administration via intramuscular injection using Zoletil. All treated animals started favipiravir treatment 2 days before infection (day −2) and received on the first day of treatment a loading dose of 200 mg/kg BID (study 1, followed by 100 mg/kg BID for 12 days) or 250 mg/kg BID (studies 2 and 3, followed by 150 and 180 mg/kg BID, respectively, for 12 days). Untreated animals did not receive any vehicle and were not anesthetized.

#### Sampling

The sampling protocol was similar to what was implemented previously in untreated NHPs [11]. Blood was collected from the femoral vein under anesthesia at days 0 (before infection on the same day), 2 or 3, 5, 7, 9 or 10, 12, 14, 17, 19, and 21 post-infection in EDTA tubes. Blood collection in treated animals was performed just before favipiravir injection, and thus the drug concentrations correspond to plasma trough concentrations.

### Data collected

#### Biochemical and hematological follow-up

Plasma levels of enzymes (ALP, ALT), creatinine, urea, and C-reactive protein (CRP) were assessed at all sampling times using a Pentra C200 Analyzer (Horiba). Blood cell counts and hemoglobin were determined from EDTA blood samples using the MS9-5s Hematology Analyzer (Melet Schloesing Laboratoires). All results were expressed as median values stratified by dosing group.

#### Molecular viral load

A synthetic RNA template, including the envelope gene region targeted by the Gibb system [16], was produced using the MEGAshortscript T7 Transcription Kit (Thermo Fisher Scientific) and quantified by spectrophotometry. EBOV genomic RNA was detected in NHP plasma samples by real-time reverse transcription PCR using the Gibb system and the GoTaq Probe 1-Step RT-qPCR Kit (Promega) following manufacturer’s instructions. Quantification was performed with reference to the standard curve obtained from serial dilutions of the standardized synthetic RNA template.

#### Infectious virus titers

Blood virus titer was determined using 12-well microplates of Vero E6 cells. Cells were incubated with serial dilutions of plasma (1 hour, 37°C), then grown in the presence of carboxymethyl cellulose (37°C, 7 days). Infectious foci were detected by incubation with an EBOV-glycoprotein-specific monoclonal antibody (provided by L. Bellanger and F. Gallais, LI2D Laboratory, CEA Marcoule, Marcoule, France), followed by phosphatase-conjugated polyclonal anti-mouse IgG and 1-Step NBT/BCIP plus Suppressor (Thermo Fisher Scientific). Virus titer was expressed as focus-forming units (ffu) per milliliter of plasma.

#### Favipiravir pharmacokinetics

Plasma aliquots were heated at 60°C for 1 hour to inactivate EBOV, then refrozen (−20°C) and transferred to another INSERM laboratory for drug concentration measurement, using a high-performance liquid chromatography procedure validated in uninfected NHPs, with a limit of quantification of 1 μg/l (see more details on the method and quality controls in [12,13]).

#### Viral genomic sequence analysis

The protocol for virus sequencing was similar to what was reported previously in untreated animals [11]. Briefly, after viral RNA extraction from plasma samples with molecular viral load higher than 3,000 copies/ml, 8 overlapping amplicons were produced using the reverse transcriptase Platinum Taq DNA Polymerase High Fidelity polymerase enzyme (Thermo Fisher Scientific) and specific primers (S1 Table). PCR products were pooled in equimolar proportions for library building. Sequencing was performed using the S5 Ion Torrent technology (Thermo Fisher Scientific) following manufacturer’s instructions. Consensus sequence was obtained after mapping of the reads on a reference (inoculum strain) using CLC Genomics Workbench software (Qiagen). A de novo contig was also produced to ensure that the consensus sequence was not affected by the reference sequence. Substitutions with a frequency higher than 1% (minor variants: variants with frequency >1% and <50%) were considered for further analysis.

Analyses included the characterization of the variants (transitions/transversions; synonymous/non-synonymous; genomic localization) and the evolution of the number of mutations over time and according to the pharmacokinetics of favipiravir and viral loads.

Of note, quantification of mutants was reported as either (i) the number of distinct genomic variant sites in a given biological sample, referred to as “number of minor variant sites,” or (ii) the volume of variant nucleotides per genome [11], taking into account the frequency of mutations at each variant site, referred to as “number of mutations per (individual viral) genome.”

Detailed protocols for extraction, amplification, sequencing, and mutation analyses are provided in S3 Text.

### Statistics

Untreated animals from the 3 experiments (*N =* 13) were pooled and treated as a single group in all statistical comparisons. The following viral kinetic parameters were calculated and are presented as median (min–max): viral load and viral titers at day 7, peak viremia, time to peak viremia, time to viral clearance, and titer clearance. Parameter values in each dosing group of favipiravir treatment were compared to values in the untreated group, and a level of significance of 0.01 was used to adjust for multiple comparisons. The impact of dose on survival time was estimated using a log-rank test, and the impact on viral kinetic parameters using a Wilcoxon rank-sum test.

Following viral kinetic model theory, the association between log viral load at day 7 (LVL) and the average trough drug concentration observed until day 7 was fitted according to the following equation:

$$LVL={LVL}_{0}-({{LVL}_{0}-LVL}_{max})\times \frac{C}{C+C_{50}^{V}}$$ (1)

where LVL_0_ is the LVL in the absence of treatment, LVL_max_ is the maximal effect that can be seen, and $C_{50}^{V}$ is the drug concentration required to achieve half the maximal effect. The same equation was used for log viral titer at day 7 (LVT):

$$LVT={LVT}_{0}-({{LVT}_{0}-LVT}_{max})\times \frac{C}{C+C_{50}^{T}}$$ (2)

with LVT_0_ the (log) viral titer at day 7 in the absence of treatment, LVT_max_ the maximal effect, and $C_{50}^{T}$ the drug concentration required to achieve half the maximal effect. Likewise, the association between the number of minor mutants at day 7 (NMM) and the average trough drug concentration observed until day 7 was fitted according to the following equation:

$$NMM={NMM}_{0}-({NMM}_{0}-{NMM}_{max})\times \frac{C}{C+C_{50}^{N}}$$ (3)

with NMM_0_ the number of minor mutants at day 7 in the absence of treatment, NMM_max_ the maximal number of mutants, and $C_{50}^{N}$ the drug concentration required to achieve half the maximal effect. To assess the significance of drug concentration, the likelihood ratio test was used, with the reference model assuming no effect of drug concentration.

All tests, fitting, and graphics were performed using R software. All *P* values were 2-tailed and were considered to be significant when <0.01 to account for test multiplicity.

### Additional data

Additional data for 13 animals are presented in S1 Text and S2 Text. The animals were part of studies 1 and 2 and followed exactly the same protocol as the animals presented in the main results: uninfected animals treated with 100 mg/kg BID (*N =* 3, study 1) or 150 mg/kg BID (*N =* 4, study 2), animals treated with 100 mg/kg BID and infected with 10 ffu (*N =* 3, study 1), and animals untreated and infected with 10 ffu (*N =* 3, study 1).

## Results

### Survival and virological response

**Fig 1:**
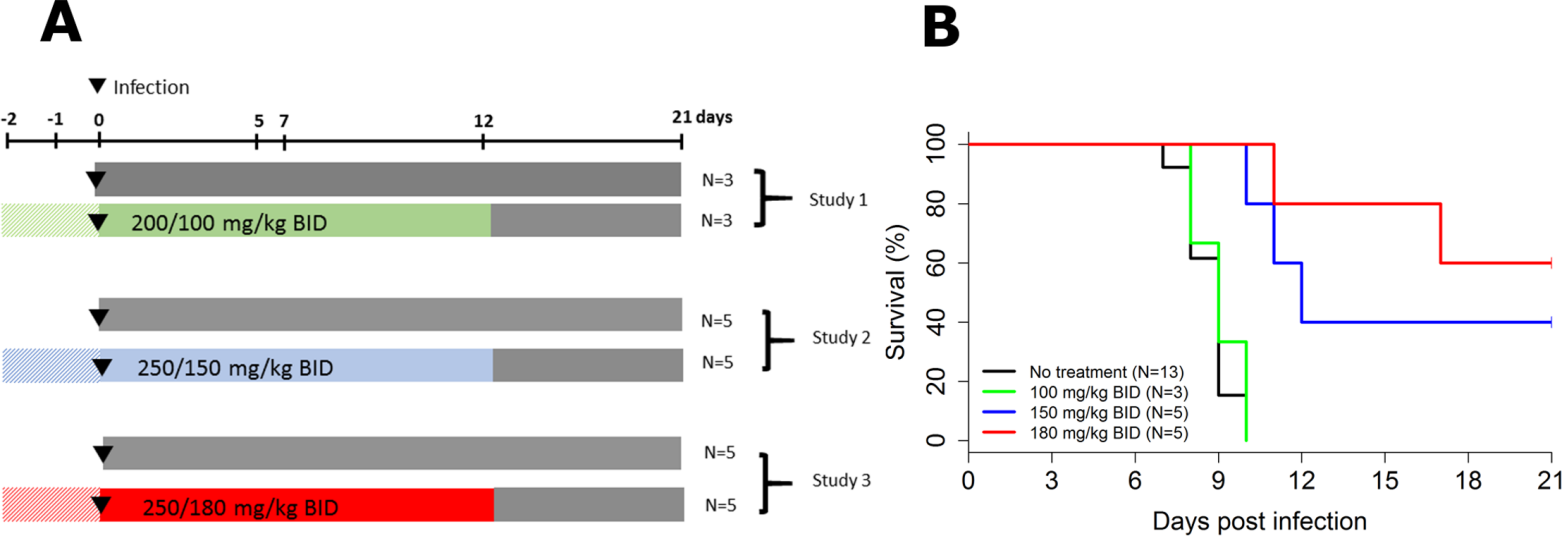
Survival according to the dosing group. (A) Study design; (B) survival curves.

Three successive experiments with increasing doses of favipiravir were performed (Fig 1A). Mauritian cynomolgus macaques were infected at day 0 with an intramuscular injection of 1,000 ffu of Ebola virus Gabon 2001 strain, as previously described [11]. All treated animals received twice daily (BID) intravenous injections of favipiravir starting with a loading dose of 200 or 250 mg/kg BID 2 days prior the infection (day −2). The treatment continued for 14 days (until day 12 post-infection [D12]) with maintenance doses of 100, 150, or 180 mg/kg BID (Fig 1A).

All untreated animals died or were euthanized between D7 and D10, with a median survival time of 9 days, consistent with our previous results [11]. Similar results were obtained in NHPs receiving 100 mg/kg BID, but 2 out of 5 (40%) and 3 out of 5 (60%) animals receiving 150 and 180 mg/kg BID, respectively, survived until the endpoint criterion (D21), and survival time was significantly increased in both groups compared to untreated animals (*P <* 0.001 in both cases, log-rank test; Fig 1B).

There was no significant decrease in median molecular peak viremia (D7) in animals receiving 100 mg/kg BID compared to untreated animals (6.9 versus 7.8 log_10_ copies/ml, *P* = 0.2). In contrast, the 2 larger doses of favipiravir led to large reductions of molecular viral load at D7, with median values of 5.9 and 4.4 log_10_ copies/ml in animals receiving 150 and 180 mg/kg BID, respectively (*P* = 0.02 and 0.003 compared to untreated animals, respectively; Table 1). Infectious viral load was also profoundly reduced by treatment, with median titers of 3.9, 2.8, and 2.0 log_10_ ffu/ml in the 100, 150, and 180 mg/kg groups, respectively, versus 6.4 log_10_ ffu/ml in untreated animals (*P* = 0.007, 0.002, and 0.008 compared to untreated animals, respectively; Table 1).

**Table 1:** Viral kinetic parameters according to the dosing group.

Treatment group	Molecular viral load at D7 (log_10_ copies/ml)	Viral titer at D7 (log_10_ ffu/ml)	Time to peak molecular viral load (days)	Peak molecular viral load (log_10_ copies/ml)	Time to clearance of molecular viral load (days)	Time to clearance of viral titer (days)
No treatment (*N =* 13)	7.8 (5.9–9.1)	6.4 (5.0–7.4)	7 (5–8)	7.9 (6.3–9.1)	—	—
100 mg/kg BID (*N =* 3)	6.9 (6.0–8.0)	3.9** (2.6–4.7)	7 (5–8)	7.1 (6.2–8)	—	—
150 mg/kg BID (*N =* 5)	5.9 (3.5–7.5)	2.8** (1.0–4.3)	10** (7–12)	7.5 (4.6–7.9)	—	12^++^ (3–14)
180 mg/kg BID (*N =* 5)	4.4** (3.5–7.0)	2.0** (1.0–5.3)	10 (5–12)	5.3** (4.9–7.3)	18^+^ (17–19)	12^+++^ (12–17)

All results provided as median value (min–max).

^+^*N =* 2.

^++^*N =* 3.

^+++^*N =* 4.

**P < 0.01 compared to untreated animals.

ffu, focus-forming units.

**Fig 2:**
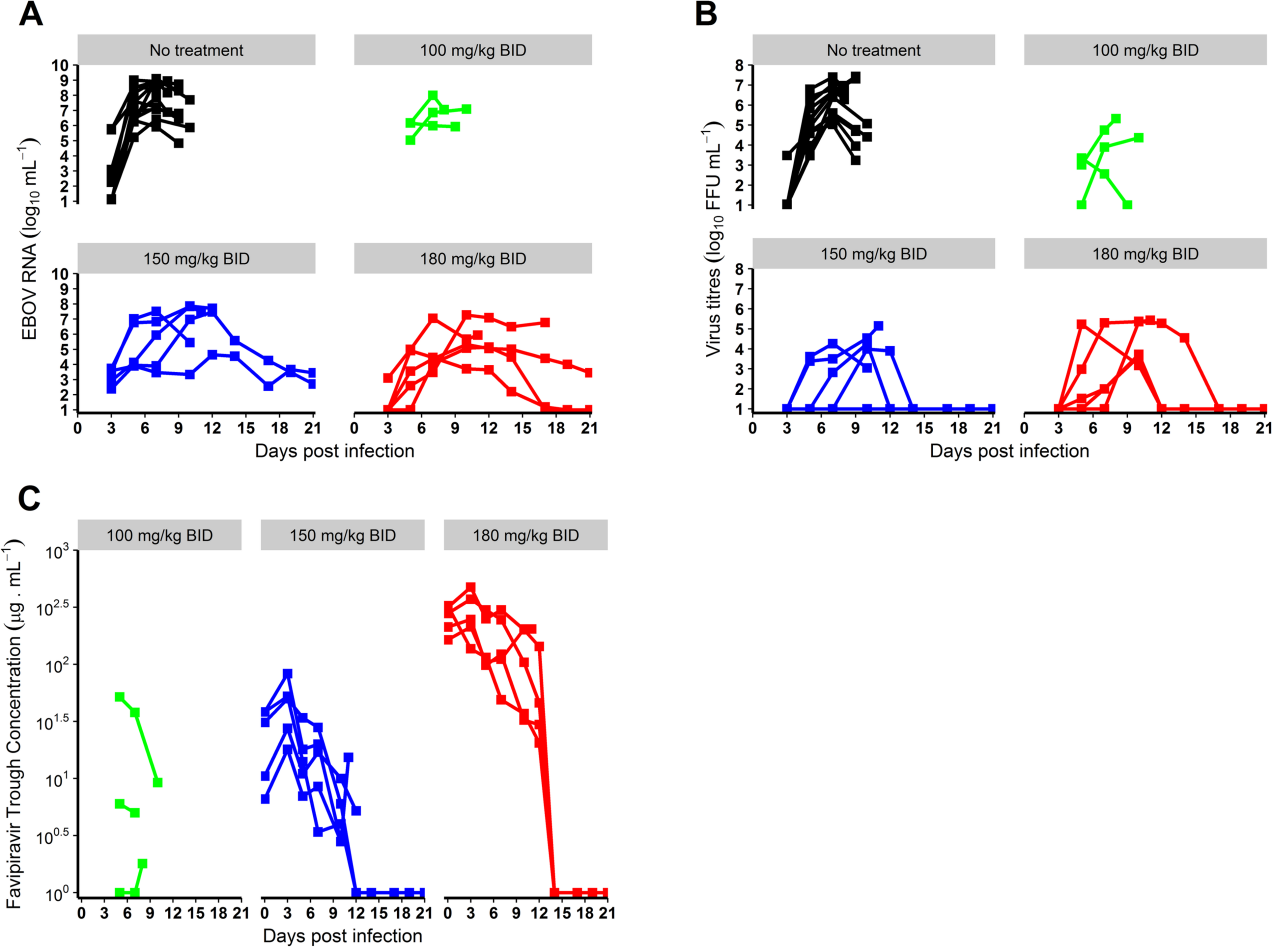
Virological and pharmacological results according to the dosing group. (A) Blood molecular viral load; (B) blood virus titer; (C) favipiravir trough concentrations. EBOV, Ebola virus; ffu, focus-forming units.

While the peak of molecular viral load occurred at D7 in the untreated and 100 mg/kg BID groups, it was delayed in animals receiving 150 or 180 mg/kg BID, with a median peak viremia achieved at D10 (Table 1; Fig 2A). Only 2 animals (receiving 180 mg/kg BID) achieved undetectable viremia, at days 17 and 19 (Fig 2A). In contrast, 3 and 4 animals receiving 150 and 180 mg/kg BID, respectively, achieved undetectable viral titers, with a median time to clearance of 12 days (Table 1; Fig 2B).

### Clinical, blood chemistry, and hematological evolution

**Fig 3:**
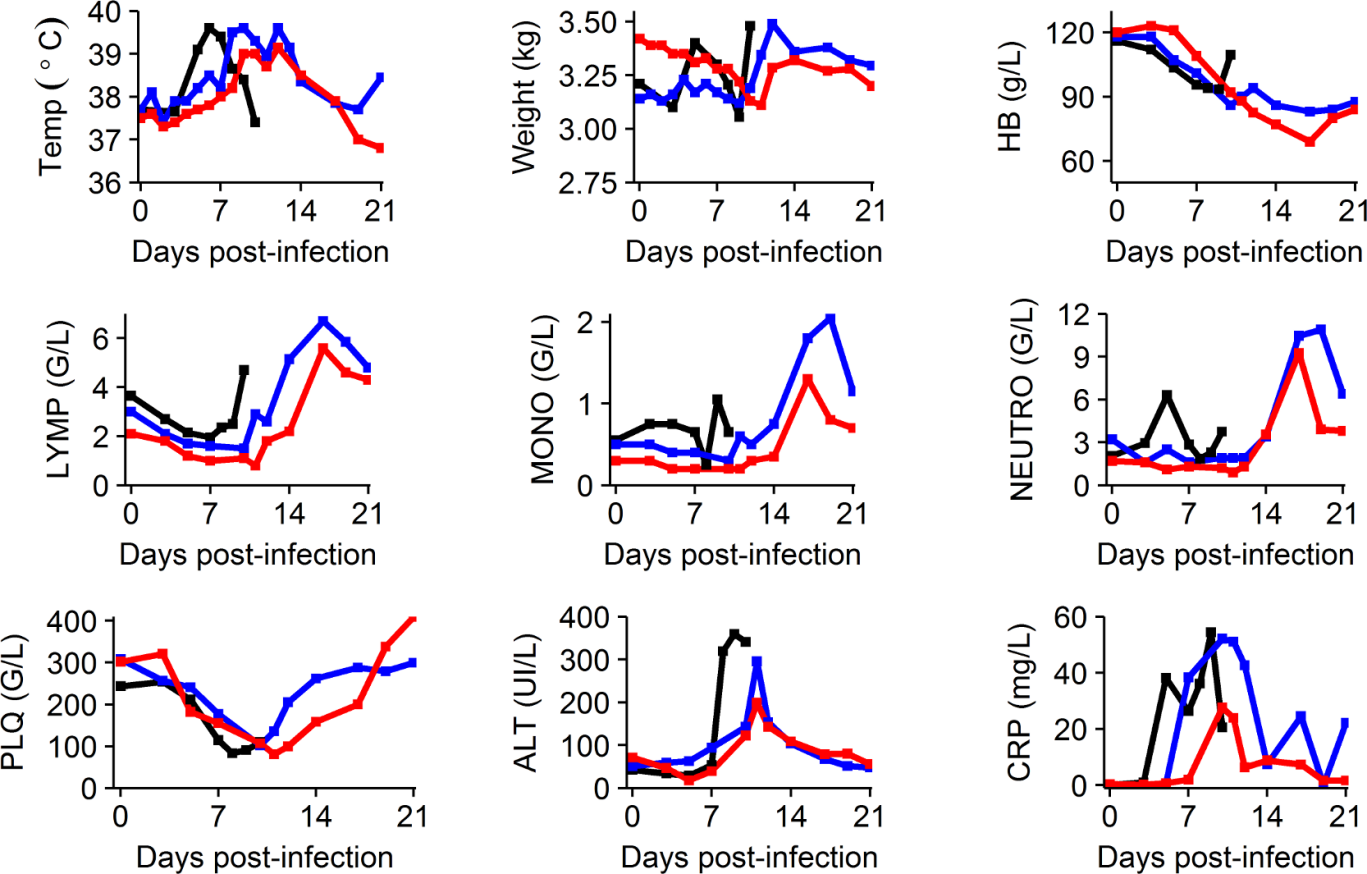
Clinical, biochemical, and hematological evolution according to the dosing group. Median values are shown for the following parameters: temperature (Temp), weight, hemoglobin (HB), lymphocyte count (LYMP), monocyte count (MONO), neutrophil count (NEUTRO), platelets (PLQ) (giga/l [G/L]), alanine aminotransferase (ALT), and C-reactive protein (CRP). Black: untreated; blue: 150 mg/kg BID; red: 180 mg/kg BID. More parameters are available in S2 Fig. Given the limited number of data available, animals treated with 100 mg/kg BID are not represented.

Clinical manifestations of EBOV infection were mostly visible after D5 and led to a rapid deterioration after D7 in the untreated and 100 mg/kg BID groups (S1 Fig). This deterioration was associated with a marked decrease in food and water intake, reduced activity, diarrhea, epistaxis, and occasional signs of cutaneous hemorrhage (petechiae restricted to the arms, legs, chest, and face). Animals treated with 150 and 180 mg/kg BID showed delayed signs of infection (S1 Fig). Consistently, ALT and CRP flares were delayed in animals receiving 150 and 180 mg/kg BID compared to untreated animals, and peak values were lower for the 150 and 180 mg/kg groups for ALT and for the 180 mg/kg group for CRP. These parameters progressively improved after the peak of viremia at D10 in surviving animals (Figs 3 and S2). Platelet and lymphocyte counts decreased initially in all groups, but a marked increase was observed in surviving animals after D10, a feature that was also observed for monocytes and neutrophils. In the 5 treated animals that survived until D21, 2 showed no sign evocative of infection at any point during follow-up and 3 showed transient signs of infection that resolved (S1 Fig).

Animals did not show severe adverse signs associated with twice daily anesthesia and treatment injection, except for a few episodes of regurgitation during injection and 1 animal presenting a wound at the site of injection, probably due to itching. In uninfected animals receiving favipiravir, clinical disease score remained lower than 5 at all times. Consistent with our previous report [10], a decrease in hemoglobin levels was observed in treated uninfected animals (20–30 g/l), but was less pronounced than in treated infected animals (30–40 g/l) (S2 Text). Other clinical, biochemical, and hematological parameters showed no significant change during follow-up in these animals.

### Effect of drug concentration on survival and virological response at day 7

**Fig 4:**
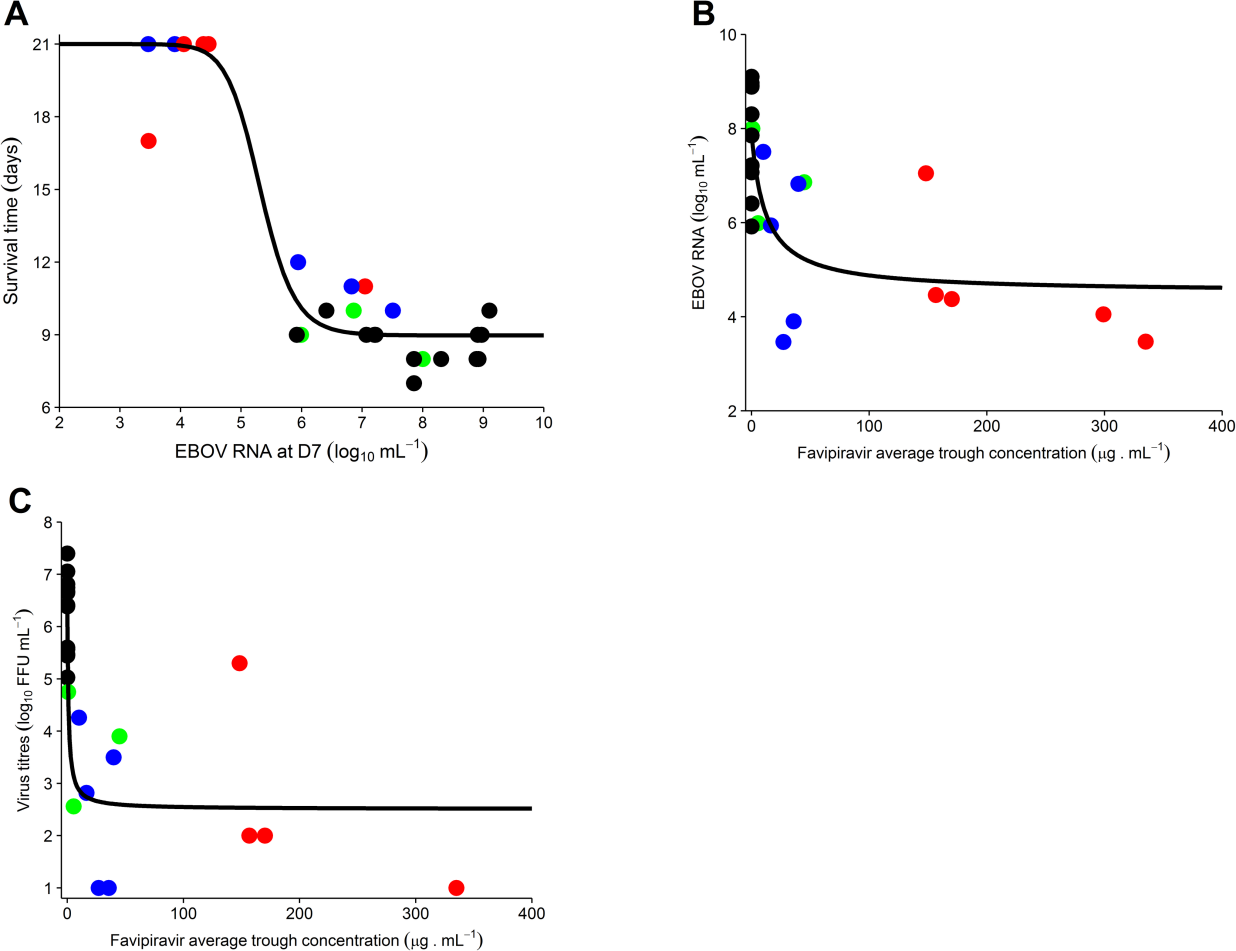
Association between average trough drug concentration until D7 and survival and virological response at D7. (A) Survival time according to molecular viral load at D7; (B) molecular viral load at D7 according to favipiravir average trough drug concentration until D7; (C) viral titer at D7 according to the average trough drug concentration until D7 (data missing for 1 animal in the 180 mg/kg BID group). The black solid line indicates the best fit to the data (see Methods). Black: untreated; green: 100 mg/kg BID; blue: 150 mg/kg BID; red: 180 mg/kg BID. EBOV, Ebola virus; ffu, focus-forming units.

Next, we investigated whether the improved responses in treated animals were linked with favipiravir plasma concentration (Fig 2C). Given that molecular viral load at D7 was strongly associated with survival (Fig 4A) and the existence of a survival bias afterwards, we focused our analysis on the relationships observed at D7. The molecular viral load at D7 was associated with favipiravir trough concentration (Fig 4B; *P <* 0.001). Favipiravir produced a maximal reduction in viral load at D7 (LVL_max_) equal to 4.5 (SE 0.7) log_10_ copies/ml. The drug trough concentration leading to half this maximal effect ($C_{50}^{V}$) was equal to 11.9 (SE 11.4) μg/ml (see Eq 1). Therefore, an average trough concentration of approximately 70 μg/ml was needed to achieve a mean viral load below 5 log_10_ copies/ml at D7, a threshold value below which all animals had an extended survival (Eq 1; Fig 4A). Similar results were observed for viral titers (*P <* 0.001), with drug concentrations over 70 μg/ml required to reduce titer down to 2.6 log_10_ ffu/ml at D7, a threshold value below which animals had an extended survival (Figs 4C and S3).

An important question is whether the drug concentrations at which an antiviral effect was observed could be achieved in humans. In EVD patients treated with oral 1,200 mg BID favipiravir (JIKI trial), median trough drug concentrations of 46.1 and 25.9 μg/ml were achieved at day 2 and 4 of treatment, respectively (Table 2). These levels are comparable with what was obtained in NHPs receiving 150 mg/kg BID, with median values of 31, 50, and 14 μg/ml at day 2, 5, and 7 of treatment, respectively. However, they remain lower than the targeted values determined above, with only 15 patients (out of 66) having at least 1 plasma drug concentration greater than 70 μg/ml, consistent with the absence of a strong signal of favipiravir antiviral activity in patients in the JIKI trial [2]. Of note, drug concentration measurements in the JIKI trial were performed in the same laboratory and using strictly identical procedures as in the present study.

**Table 2:** Drug concentrations observed in nonhuman primates compared to what was observed in humans in the JIKI trial [12].

Days post-treatment initiation	Nonhuman primates				Humans—JIKI trial	
	Days post-infection	Drug concentration by maintenance dose			Days from symptom onset	Drug concentration with maintenance dose 1,200 mg BID
		100 mg/kg BID (*N =* 3)	150 mg/kg BID (*N =* 5)	180 mg/kg BID (*N =* 5)		
2	0	—	31.0 (6.6–38.3)	279.2 (164.0–326.0)	7 (3–16)	46.1 (2.3–106.9) (*N =* 44)
4	2	—	—	—	10 (5–20)	25.9 (0–173.2) (*N =* 50)
5	3	—	50.0 (18.0–83.0)	247.5 (137.0–474.2)	—	—
7	5	6.0 (0.4–51.8)	14.0 (7.0–34.0)	115.0 (98.3–300.0)	—	—
9	7	5.0 (0.0–37.9)	17.0 (3.4–28.0)	122.5 (49.0–299.2)	—	—

All results provided as median value (min–max).

Because it has been suggested that infection could affect favipiravir pharmacokinetics [17], a comparison of drug pharmacokinetics in infected and uninfected animals receiving the same dosing regimen was performed. It revealed no significant difference between the two groups (S2 Text).

### Mutagenesis

The impact of favipiravir treatment on genomic evolution of EBOV was estimated by viral genomic direct deep sequencing (i.e., without virus isolation in cell culture) of 92 plasma specimens sampled longitudinally. The mean sequencing coverage was 26,600 reads per genomic position, and substitutions with a frequency higher than 1% were considered for further analysis. For comparison purposes, 74 samples obtained at D5, D7, and D8–D10 were analyzed. These included 38 samples from untreated animals (D5, *N =* 13; D7, *N =* 13; D8–D10, *N =* 12) and 36 from treated animals (100 mg/kg BID: D5, *N =* 3; D7, *N =* 3; D8–D10, *N =* 3; 150 mg/kg BID: D5, *N =* 5; D7, *N =* 4; D8–D10, *N =* 4; 180 mg/kg BID: D5, *N =* 4; D7, *N =* 5; D8–D10, *N =* 5). Additional sequencing was performed for 18 samples at D3 and in the D11–D21 period. GenBank numbers and raw data references are provided in S2 Table, and description of minor variant sites is presented in S3 Table.

With reference to the inoculum strain (GenBank KY471124), major variants (i.e., mutations identified in more than half of the corresponding reads in a given sample) were rare. They were observed in only 3 treated animals, leading to a total of 12 mutations (D5: *N =* 3; D7: *N =* 6; D10: *N =* 3; S4 Table), including only 3 with a frequency over 80%. These mutations were essentially transitions (10/12), and all altered sites were in coding regions with a majority of non-synonymous mutations (10/12), a pattern compatible with an ongoing selection of adaptive mutants. However, no mutation in the polymerase gene was observed, and there was therefore no evidence for the emergence of mutants with a favipiravir resistance profile.

**Fig 5:**
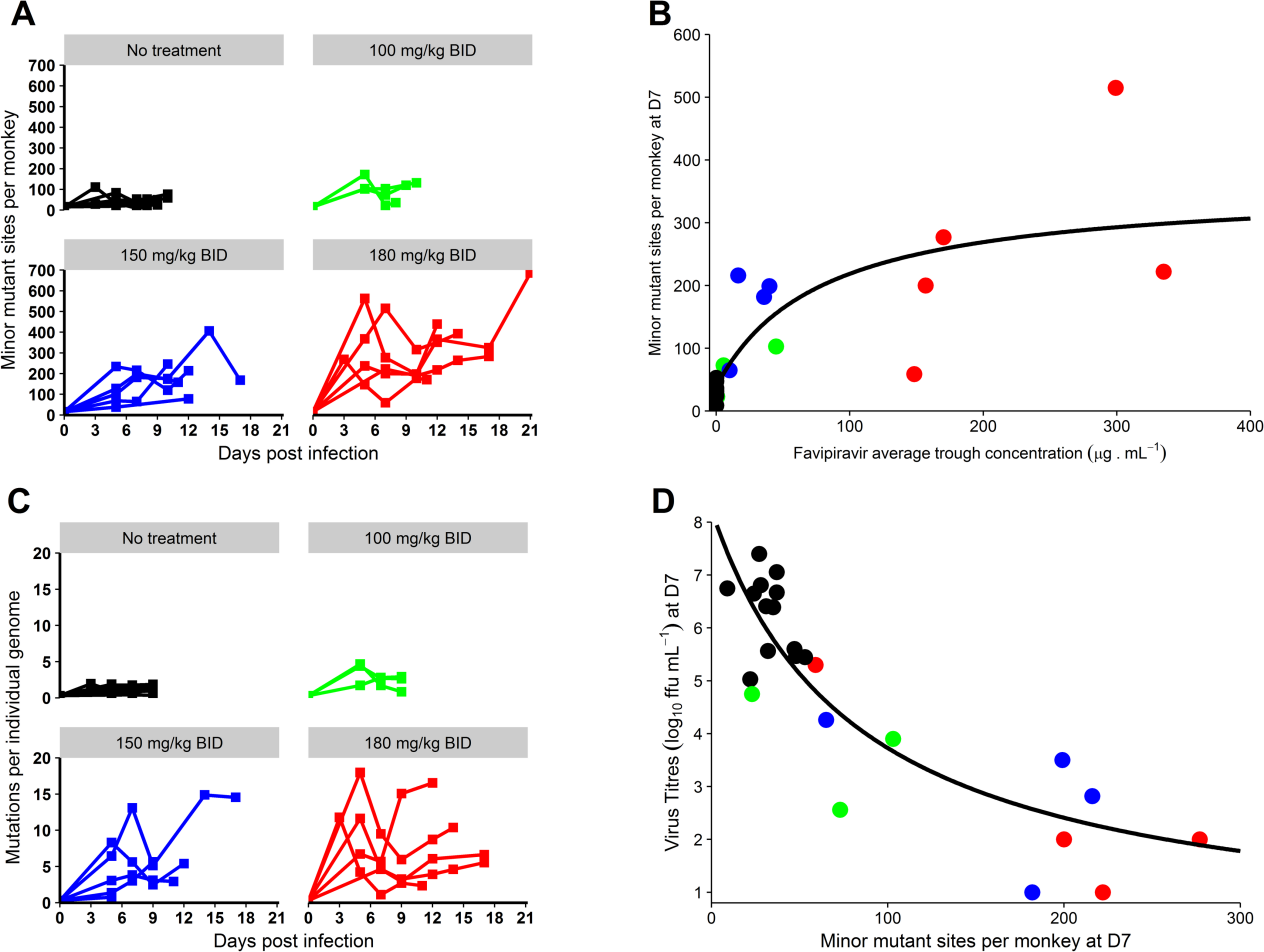
Genomic evolution of the virus according to the dosing group. (A) Evolution of the number of minor mutant sites over time; (B) number of minor mutant sites at D7 according to average favipiravir trough concentration until D7; (C) evolution of the number of mutations per individual genome over time; (D) viral titers at D7 according to the number of minor mutant sites at D7 (data missing for 1 animal in the 180 mg/kg BID group). The black solid line indicates the best fit to the data (see Methods). Black: untreated; green: 100 mg/kg BID; blue: 150 mg/kg BID; red: 180 mg/kg BID. ffu, focus-forming units.

A completely different picture was observed for minor variants (variants with frequency >1% and <50%), whose number increased in a dose-dependent manner. This included both the number of minor mutant sites per monkey (Fig 5A and 5B) and the number of mutations per individual viral genome (Fig 5C; Table 3). This accumulation of mutations was drug concentration dependent (*P <* 0.001; Fig 5B) and was associated with lower levels of plasma infectious viral particles, in particular at D7 (Fig 5D).

**Table 3:** Median genetic distances and infectivity ratio values according to time and dosing group.

Days post-infection	Dosing group	Median genetic distance* (× 10^−5^)	Infectivity ratio** (× 10^−4^)
—	Inoculum	1.59	—
5	No treatment	6.73 (2.75–9.94)	32.4 (0.14–552)
	150 mg/kg BID	16.20 (4.02–44.0)	7.42 (3.91–12.5)
	180 mg/kg BID	48.60 (22.4–95.3)	62.1 (4.02–251)
7	No treatment	4.16 (3.41–9.32)	90.8 (0.23–4,410)
	150 mg/kg BID	2.50 (1.60–6.94)	7.57 (4.73–34.2)
	180 mg/kg BID	2.46 (0.58–5.04)	41.8 (34.5–179)
10	No treatment	6.69 (4.46–9.87)	175 (43.3–8,740)
	150 mg/kg BID	2.18 (1.31–2.99)	10.4 (2.50–46.3)
	180 mg/kg BID	1.73 (1.43–7.99)	253 (123–8,060)

All results provided as median value (min–max).

*The median genetic distance to the inoculum sequence was calculated as the median number of mutations per genome (see Fig 4C) divided by Ebola virus genome length.

**The infectivity ratio was calculated as the ratio of infectious viral load over molecular viral load.

**Fig 6:**
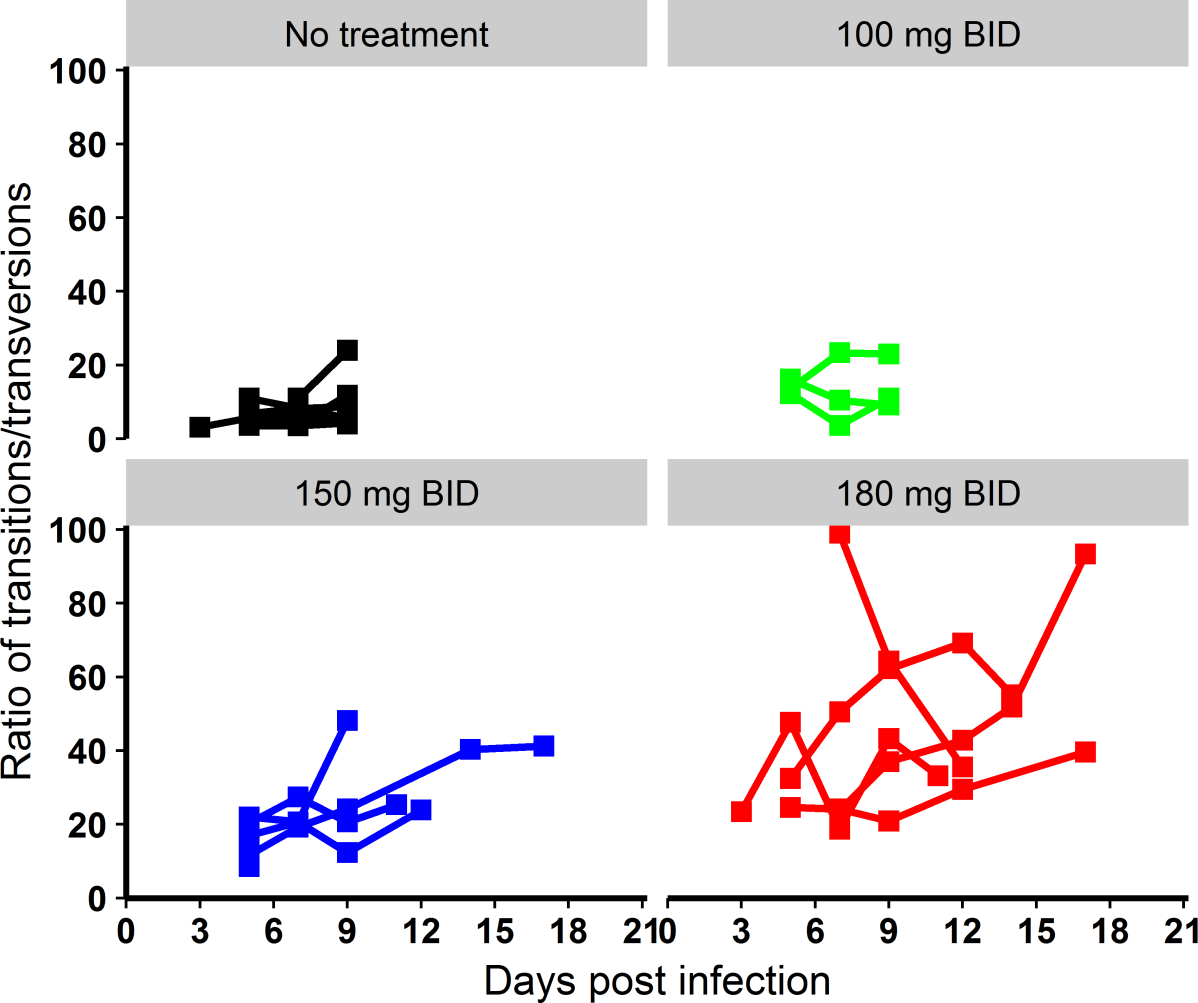
Evolution of the transition/transversion ratio overtime. Black: untreated; green: 100 mg/kg BID; blue: 150 mg/kg BID; red: 180 mg/kg BID.

Next, the analysis of mutagenesis patterns revealed that favipiravir preferentially induced transition mutations, with a marked dose-dependent increase of the transition/transversion ratio (Fig 6). The pattern of transition substitutions showed the predominance of C→T and G→A transitions (S4 Fig).

The mutagenesis mechanism of favipiravir is not anticipated to affect differently the 3 positions of the codon. A random distribution of mutations over the codon positions would lead to a proportion of non-synonymous mutations in the EBOV open reading frames of approximately 78%, close to the value observed in the inoculum strain (73%), issued from propagation onto cell culture (S5 Table). A lower proportion of non-synonymous mutations was observed in both untreated and treated animals and decreased overtime to reach 51%–54% at D8–D10, indicating a negative selection of variants with non-synonymous deleterious mutations. Because favipiravir is expected to have produced a large number of such non-synonymous mutants, the negative selection of these mutants contributed to the decrease of viral load and therefore to the antiviral activity of the molecule.

## Discussion

In summary, we demonstrated that the polymerase inhibitor favipiravir is an effective treatment against EBOV in a lethal NHP model [11]. When treatment of Mauritian cynomolgus macaques was initiated 2 days prior to infection, favipiravir administered intravenously reduced viremia in a concentration-dependent manner, with an increase in mutagenesis and a reduction of virus infectivity. Survival rates of 40% and 60% were observed at doses of 150 and 180 mg/kg BID, respectively.

Our first experiment, using a dose of 100 mg/kg BID, showed no impact on survival and lower plasma drug concentrations than expected, which was confirmed in a PK study conducted afterwards [13]. Based on these findings, we performed detailed PK analyses in uninfected NHPs at doses of 150 and 180 mg/kg BID [13]. These analyses revealed that favipiravir had complex nonlinear pharmacokinetics due to a concentration-dependent inhibition of aldehyde oxidase, the main enzyme involved in favipiravir metabolism [13]. By combining these data with a viral dynamic analysis (see S1 Text), we could identify that doses of 150 and 180 mg/kg BID were both tolerable in NHPs and had the potential to largely reduce viremia in NHPs at D7. Our results confirmed this prediction, with median viral load at D7 reduced by 2 and 3 logs in animals treated with 150 and 180 mg/kg BID, respectively, compared to untreated animals, and identified a target plasma trough drug concentration to generate this effect of about 70–80 μg/ml.

This antiviral effect is likely mediated in part by an increase of EBOV mutagenesis, consistent with previous in vitro observations in the context of influenza virus [18], hepatitis C virus [19], foot-and-mouth disease virus [20], and West Nile virus [21]. The mutagenesis patterns revealed that favipiravir preferentially induced transition mutations, with a predominance of C→T and G→A transitions, as previously reported for influenza and hepatitis C virus [19] and reflecting the possible competition for integration into the nascent RNA chain between the favipiravir metabolite T-705-4-ribofuranosyl-5′-triphosphate (T-705-RTP) and GTP [22]. The mechanism of appearance of mutations would potentially imply the copying of both the negative and positive strands of the genome by the virus polymerase, as summarized in S5 Fig. Such accumulation of mutations can reduce the potential of viral particles to produce a full infectious cycle when the virus mutation rate goes beyond the biological tolerance threshold, causing lethal mutagenesis by a phenomenon commonly referred to as “error catastrophe.” In addition, favipiravir has been shown to act as a chain terminator [22], which can constitute an independent antiviral mechanism, but may also be part of the mutagenic process. This is, to our knowledge, the first report of an antiviral drug leading to error catastrophe in NHPs and suggests that mutagenesis could be a valuable marker of antiviral activity.

There are currently very few examples of human acute infections by RNA viruses in which antiviral therapeutics have proven to be effective, the iconic example remaining the treatment of influenza A by inhibitors of the virus neuraminidase [23]. In the context of EBOV infection, our results, together with those obtained with GS-5734 [24], demonstrate that an antiviral drug may prevent death in an otherwise fully lethal model. In the case of favipiravir, our results suggest the following chain of events: the early initiation of treatment reduces viral load and increases the number of low fitness mutants, which reduces the severity of the disease and possibly its immunosuppressive effect [25]. This in turn allows for an effective activation of the immune response, which allows clearance of the virus, as suggested by the increase in lymphocytes and the absence of detectable infectious virus at the study endpoint (D21) in surviving animals.

The biosafety environment of a BSL4 laboratory introduces a number of methodological and technical limitations, in particular the difficulty of conducting blind studies [26]. In order to minimize potential bias and inherent subjectivity, an exhaustive follow-up of the animals was performed daily, which included assessment of disease signs by video surveillance along with a detailed clinical score (S3 Fig). Of note, all 5 surviving animals had no sign evocative of infection at the study endpoint (D21), supporting the fact that infection was resolved at the end of the study. The BSL4 environment also limited pharmacological analysis, and our analysis of the concentration–effect relationship of favipiravir relied on plasma parent concentration and not on the active, intracellular metabolite favipiravir ribosyl triphosphate [27]. Although studies exploring favipiravir diffusion will be needed to assess intracellular levels, the half-life of the intracellular triphosphate metabolite is short and in the range of 2–6 hours [28], supporting the use of plasma pharmacokinetics as a relevant marker of drug exposition.

We identified that a large viral load reduction was associated with a plasma trough drug concentration of 70–80 μg/ml. This target drug concentration is larger than what was observed in EVD patients treated with oral 1,200 mg BID favipiravir (JIKI trial), who achieved median trough drug concentrations of 46.1 and 25.9 μg/ml at day 2 and 4 of treatment, respectively [12]. This therefore suggests that the dose used in the JIKI trial is unlikely to be sufficient to achieve antiviral efficacy, and is consistent with the absence of a strong signal of antiviral efficacy in this trial. Although the good tolerance of favipiravir in EVD patients [2,29] is a favorable signal towards the possibility of increasing the doses, PK and tolerance studies at relevant doses for EVD treatment will be needed to determine the appropriate dosing regimen for favipiravir.

Although these results suggest that a prophylactic use of favipiravir in humans may be effective, they will need to be complemented by post-exposure studies in NHPs to optimize the future use of favipiravir during acute infection. Yet, the generalization of these results will remain limited by the fact that treatment in humans, at least until now, has mostly been initiated several days after symptom onset (and even more days after infection), when high levels of viral replication are already present. For instance, in the JIKI trial, the median time from symptom onset to treatment initiation was 4 days [12]. Likewise, a retrospective cohort study on several hundreds of patients in Guinea estimated the mean time between symptom onset and hospitalization to be 5 days, a timing corresponding to peak viremia [30]. Thus, despite these encouraging results and those obtained with drugs showing efficacy in NHPs when administered up to 3 days after infection [15,24,31], it is likely that treatment during acute infection in humans will not rely on a unique drug, but will require combination therapy, administered as early as possible, to maximize antiviral efficacy. In addition to its use in prophylactic and acute infection settings, antiviral therapy may also have a role after clinical recovery, to clear the virus from immune sanctuaries (e.g., semen, eye, central neurological system [32–34]) and prevent relapse and dissemination.

There are no approved vaccines against EVD, although several vaccine trials have shown the capability of vaccines to elicit a sustained immune response [35–37]. Only 1 study, using a vesicular-stomatitis-virus-based vaccine, evaluated vaccine efficacy in contacts and contacts of contacts of confirmed EVD cases (“ring strategy”) and showed a high level of protection in individuals who were immediately vaccinated. Whether antivirals and vaccines can be combined to optimize protection against EBOV in post-exposure prophylaxis will need to be investigated, and may require specific attention in the case of replication-competent viruses.

Beside its activity against EBOV, favipiravir has a broad antiviral activity against RNA viruses and shows in vivo activity against several hemorrhagic fever viruses, such as Lassa, Marburg, and Crimean–Congo hemorrhagic fever viruses, in mice models [38,39]. Therefore, the use of high doses of the drug would allow considering a large number of these pathogens as potential therapeutic targets [13,40], and the results found here on the drug’s mechanism of action and pharmacokinetics may be relevant for designing favipiravir-based therapeutic protocols against these pathogens.

In summary, our results suggest that favipiravir may be an effective antiviral against EBOV that likely relies on RNA chain termination and error catastrophe. These results, together with previous data collected on tolerance and pharmacokinetics in both NHPs and humans, are likely to be transposable to other emerging or reemerging viral pathogens. This study, therefore, supports a potential role for high doses of favipiravir for future interventions in patients with EVD.

## Supporting information

S1 Fig Clinical scores. Green and blue indicate scores ≤5 and >5, respectively. Orange indicates that the animal was found dead, and red indicates that euthanasia was performed the same day. The individual CBD021 stopped eating at between D0 and D3 and ate normally afterwards, which explains the unusually high score at D3 and normal values afterwards.
(TIF)

S2 Fig Median evolution of the following clinical, biochemical, and hematological parameters by treatment group: aspartate aminotransferase (AST), alkaline phosphatase (ALP), bilirubin (TBIL), creatine kinase (CK), urea (UREA), and creatinine (CREAT). Black: untreated; blue: 150 mg/kg BID; red: 180 mg/kg BID. (TIF)

S3 Fig Survival time according to infectious viral load titer at day 7. Black: untreated; green: 100 mg/kg BID; blue: 150 mg/kg BID; red: 180 mg/kg BID.
(TIF)

S4 Fig Patterns of transition substitutions by treatment group over time. (TIF)

S5 Fig Possible substitution mechanism based on competition between favipiravir ribosyl triphosphate (FTP) and GTP. (TIF)

S1 Table Positions and sequences of primers used to amplify Ebola virus in monkey sera and inoculum. Positions in reference to KC242800 Gabon 2002 sequence (see S3 Text).
(DOCX)

S2 Table GenBank number and raw data reference for each sample sequenced in this study. (XLSX)

S3 Table Individual description of minor variants by monkey. No information is provided concerning the nature (synonymous/non-synonymous) of the variants between the positions 6924 and 7133. Three open reading frames constitute this part of the ORF. S, synonymous; NS, non-synonymous.
(XLSX)

S4 Table Ebola virus major variants observed in monkey sera. S, synonymous; NS, non-synonymous. (DOCX)

S5 Table Proportion of non-synonymous variant sites over time in the different treatment groups. The number of synonymous and non-synonymous variants was considered excluding the part of the GP gene that encodes several ORFs. Proportion of non-synonymous variants expected under a regimen of random distribution: 78%.
(DOCX)

S6 Table Individual molecular and infectious viral load data. (XLSX)

S7 Table Individual pharmacokinetic data. (XLSX)

S8 Table Individual data on number of minor mutant sites. (XLSX)

S1 Text Determination of high dose of favipiravir using a viral kinetic model. (DOCX)

S2 Text Drug concentrations in infected and uninfected nonhuman primates. (DOCX)

S3 Text Genomic analysis. (DOCX)

S4 Text Excerpt of the funding document submitted to the European Commission in 2014 describing the study protocol. (PDF)

S5 Text ARRIVE checklist. (PDF)

S6 Text Data file description. (DOCX)

## Abbreviations

BID: twice a day
BSL4: biosafety level 4
CRP: C-reactive protein
D[number]: day [number] post-infection
EBOV: Ebola virus
EC_50_: half maximal effective concentration
EVD: Ebola virus disease
ffu: focus-forming units
NHP: nonhuman primate
PK: pharmacokinetic
WHO: World Health Organization
