# Supplementary figures and images for: Antiviral efficacy of favipiravir against Ebola virus: A translational study in cynomolgus macaques
Source: PLoS Med. 2018 Mar 27;15(3):e1002535. doi: 10.1371/journal.pmed.1002535 (PMC5870946; doi:10.1371/journal.pmed.1002535)

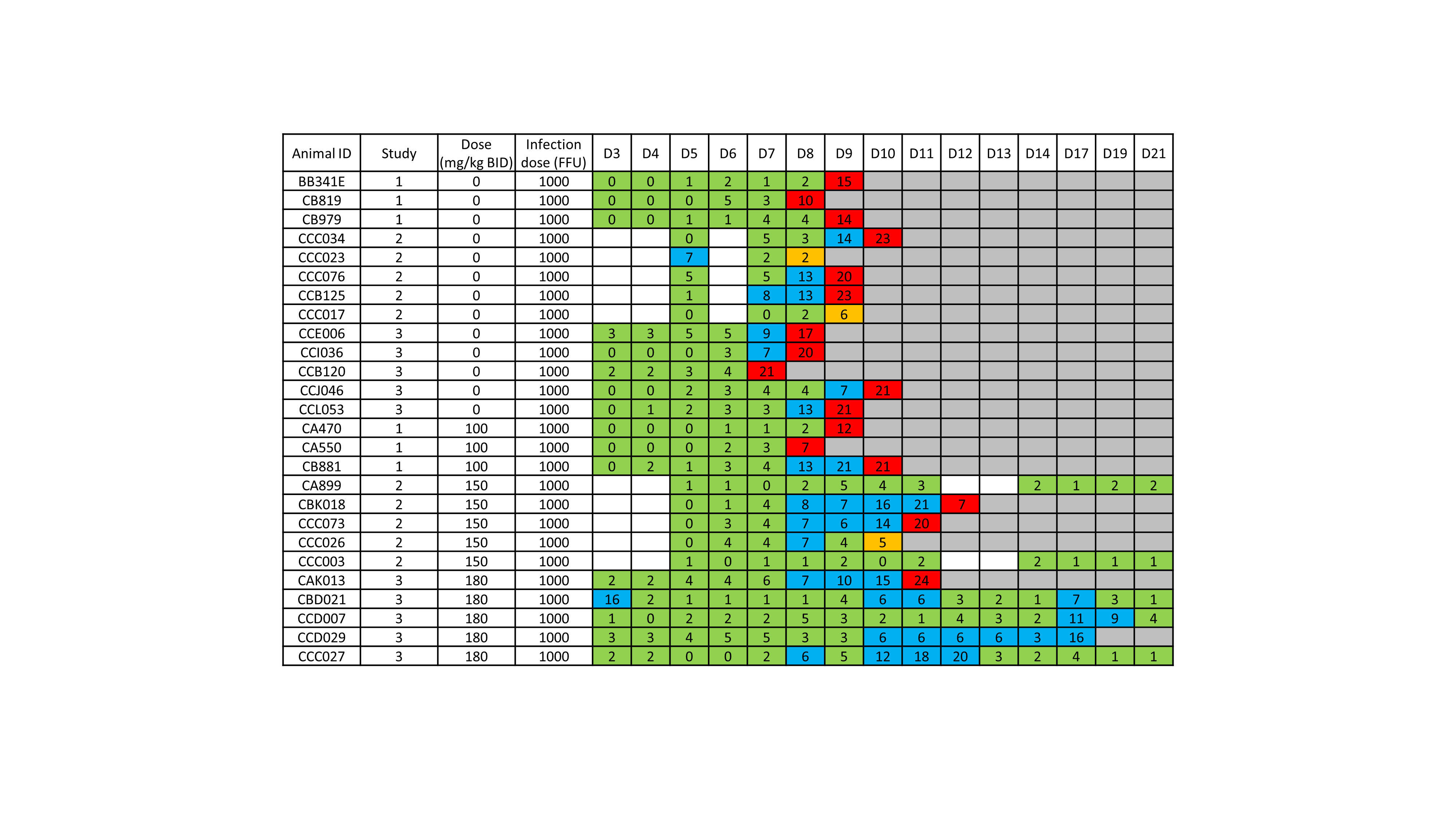

Supplement: S1 Fig — Green and blue indicate scores ≤5 and >5, respectively. Orange indicates that the animal was found dead, and red indicates that euthanasia was performed the same day. The individual CBD021 stopped eating at between D0 and D3 and ate normally afterwards, which explains the unusually high score at D3 and normal values afterwards. (TIF) [file pmed.1002535.s001.tif]

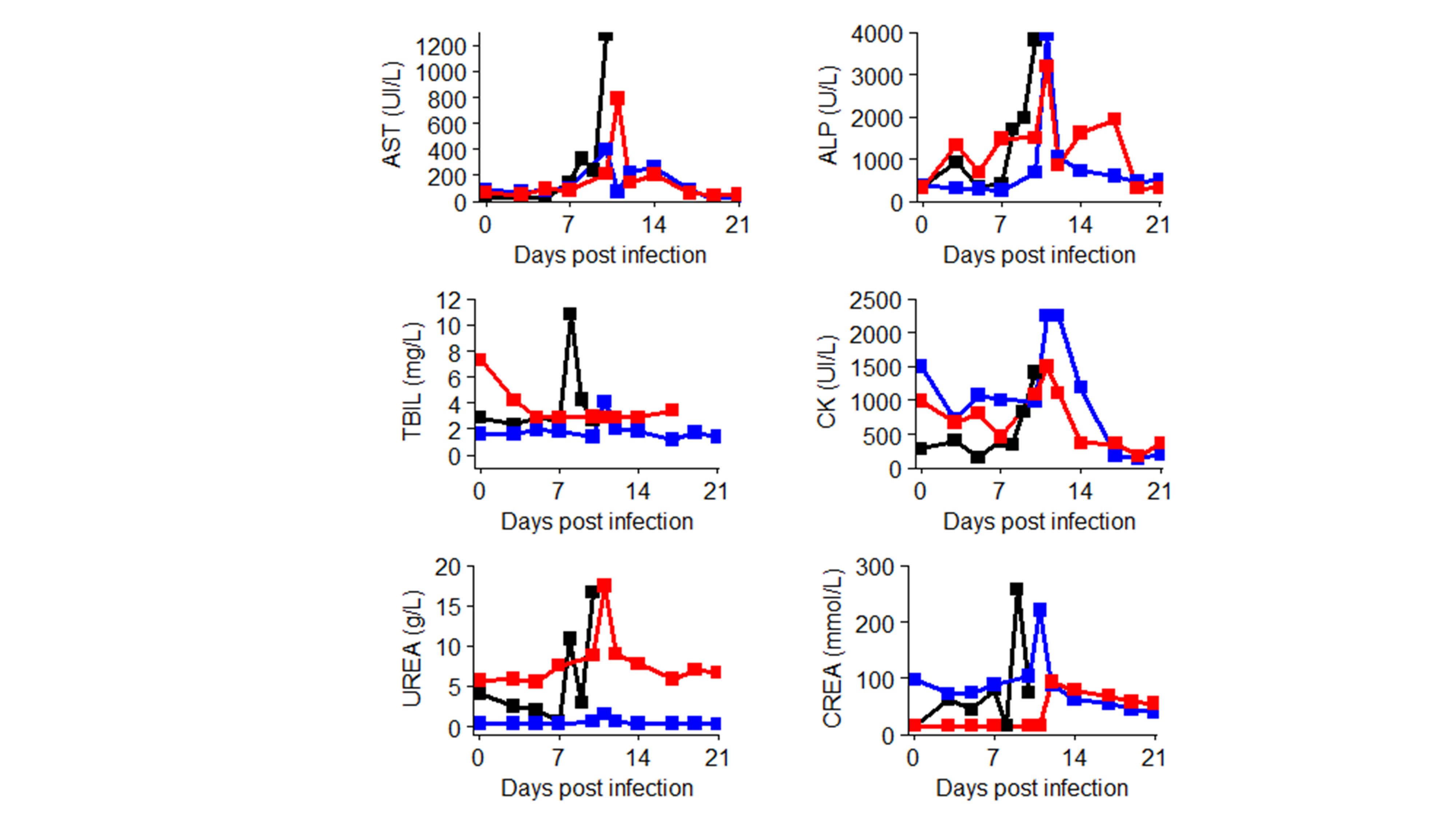

Supplement: S2 Fig — (TIF) [file pmed.1002535.s002.tif]

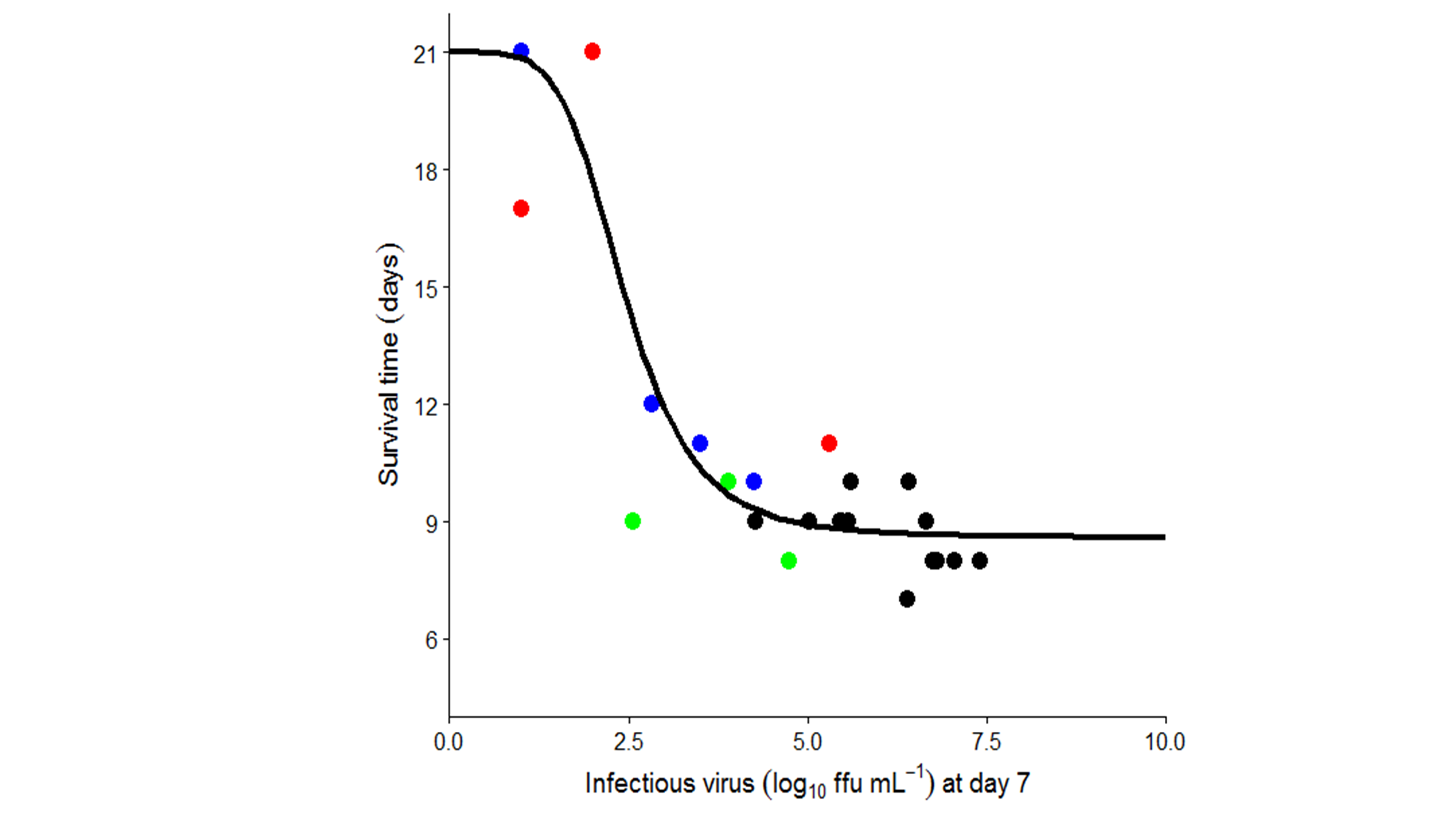

Supplement: S3 Fig — Black: untreated; green: 100 mg/kg BID; blue: 150 mg/kg BID; red: 180 mg/kg BID. (TIF) [file pmed.1002535.s003.tif]

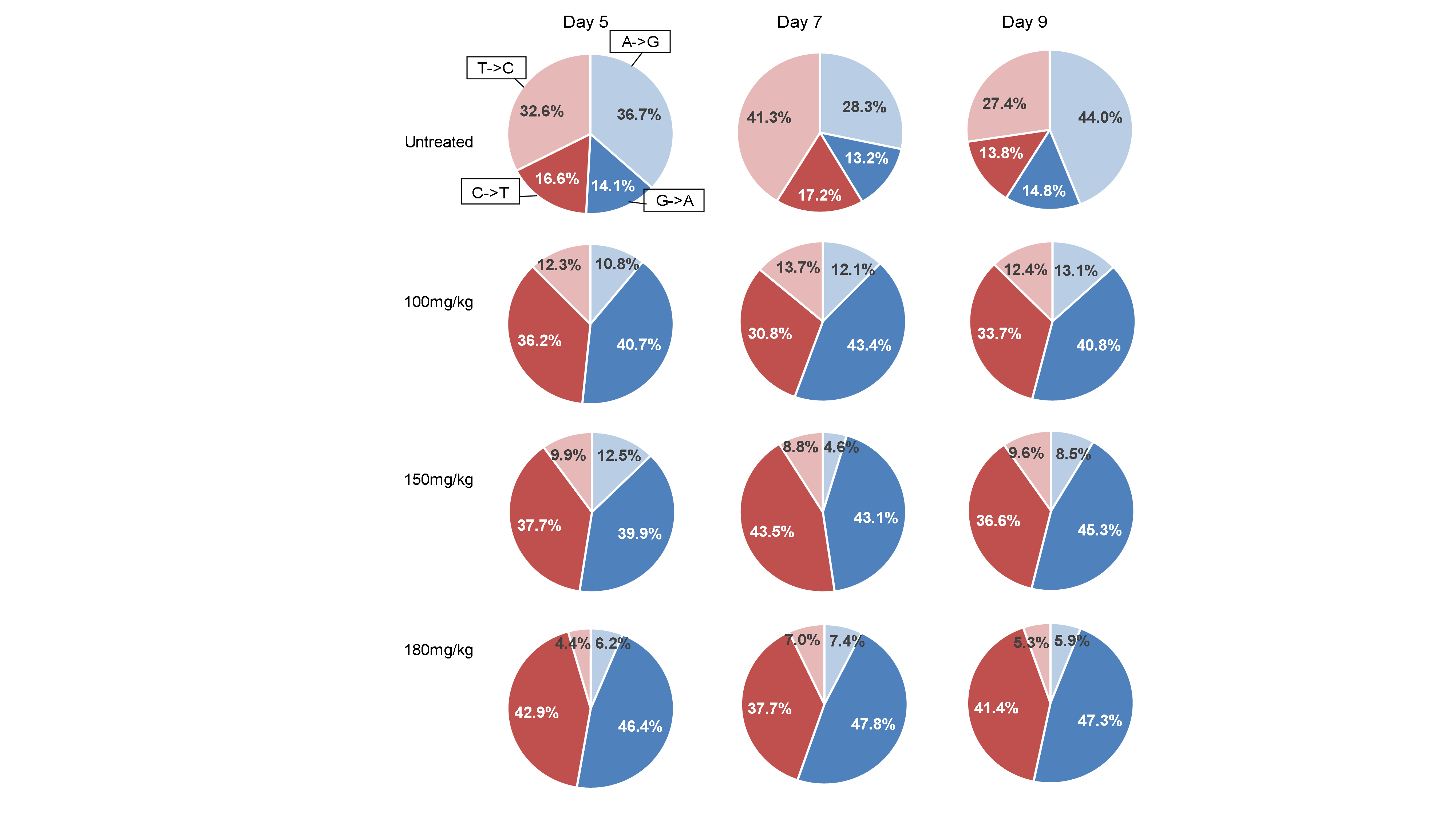

Supplement: S4 Fig — (TIF) [file pmed.1002535.s004.tif]

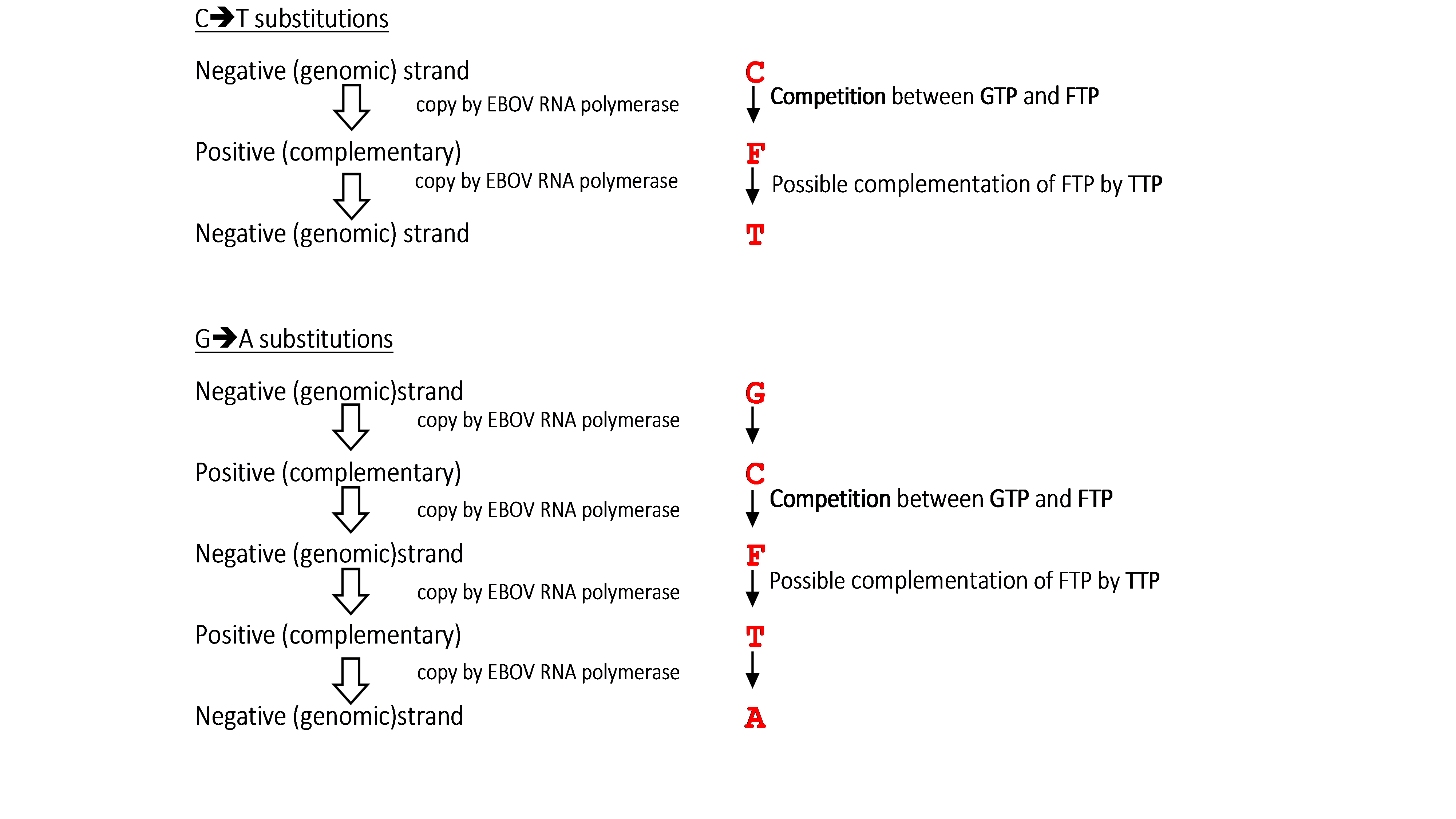

Supplement: S5 Fig — (TIF) [file pmed.1002535.s005.tif]

**S4 Table.** EBOV major variants observed in monkey sera.

S: Synonymous ; NS: Non Synonymous;
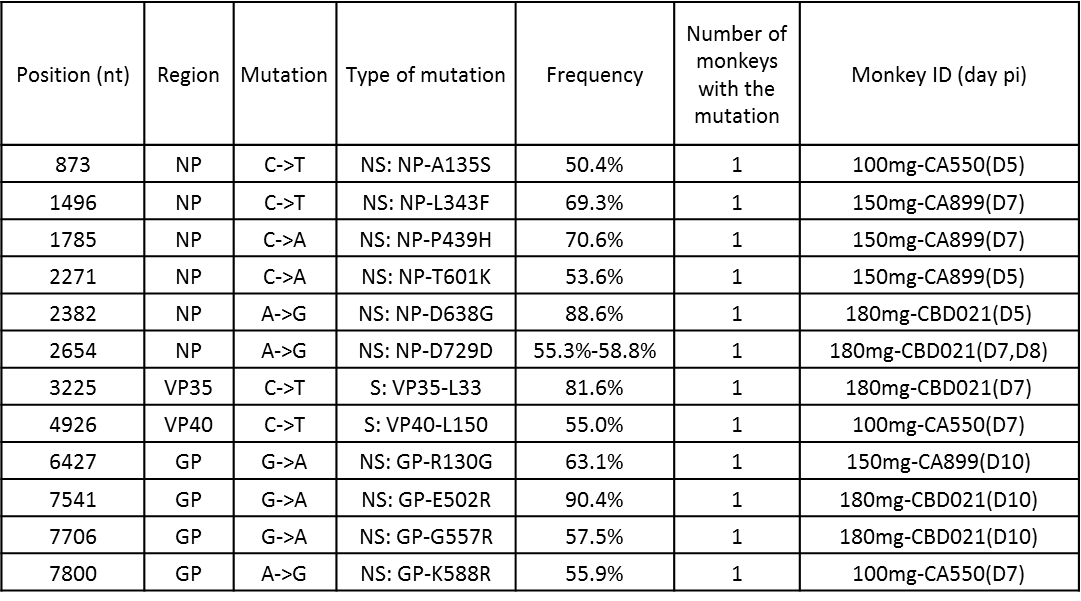

Supplement: S4 Table — (DOCX) [file pmed.1002535.s009.docx]
