# Supplementary material for: Antiviral efficacy of favipiravir against Ebola virus: A translational study in cynomolgus macaques
Source: PLoS Med. 2018 Mar 27;15(3):e1002535. doi: 10.1371/journal.pmed.1002535 (PMC5870946; doi:10.1371/journal.pmed.1002535)
Supplement: S1 Table — Positions in reference to KC242800 Gabon 2002 sequence (see S3 Text). (DOCX) [file pmed.1002535.s006.docx]

**S1 Table**: Positions and sequences of primers used to amplify EBOV in monkey sera and inoculum. Positions in reference to KC242800 Gabon 2002 sequence (see S3 text).

| Positions | Sequences |  |
| --- | --- | --- |
| 1 | CGGACACACAAAAAGAAAGAAGA | Sens 1 |
| 3333 | CCGTTTGGGTTTGACTGTTGCG | Reverse 1 |
| 2479 | GATGAAGGATGAGCCTGTAGTT | Sens 2 |
| 4872 | CAGCCGATTGACTCTGACAAG | Reverse 2 |
| 4568 | CAACAGCAATACAGGCTTCCTG | Sens 3 |
| 7552 | GTTGCATTTGGGTTGAGCATTG | Reverse 3 |
| 7077 | CCTCTGCAATGGTTCAAGTGC | Sens 4 |
| 10668 | CTGCTAAGGGTTCAATCAAAGACTGG | Reverse 4 |
| 10465 | TATTGGGCTGGTATTGAGTTTGATG | Sens 5 |
| 12244 | CAATGTGTGTATGCCCTTGAAC | Reverse 5 |
| 11773 | GATGTACCAGTGGCGACATTG | Sens 6 |
| 14645 | CGACTCATAACAGGAGTTGATG | Reverse 6 |
| 14318 | CCTTCTTGAATCCTGAGAAATG | Sens 7 |
| 16710 | ACTGGAACGACGGACCCTGG | Reverse 7 |
| 16082 | ATCTACCACATCGCTCATTGC | Sens 8 |
| 18895 | GTGTGCGACCATTTTTCCAGG | Reverse 8 |

For some virus samples, amplification with the classical primers failed, secondary couple of primers (call sens ‘ and reverse ‘) were used.

| Position | Sequences |  |  |
| --- | --- | --- | --- |
| 1 | CGGACACACAAAAAGAAAGAAGA | Sens 1 | Fragment 1 |
| 1784 | CCTGGCCATCAAGATGATGATC | Reverse 1' |  |
| 1921 | GTCCTCGTCTAGATCGAATAGG | Sens 1' |  |
| 3333 | CCGTTTGGGTTTGACTGTTGCG | Reverse 1 |  |
| 7223 | CTTGACATCTCTGAGGCAACTC | Sens 4' | Fragment 4 |
| 10626 | CCTGCTGCAAGGATGACTCTC | Reverse 4' |  |
| 14318 | CCTTCTTGAATCCTGAGAAATG | Sens 7 | Fragment 7 |
| 15067 | GACTGCCACTGACACGAATGG | Reverse 7' |  |
| 14965 | GGAGCCACACTCCCATGTATG | Sens 7' |  |
| 16710 | ACTGGAACGACGGACCCTGG | Reverse 7 |  |
| 16082 | ATCTACCACATCGCTCATTGC | Sens 8 | Fragment 8 |
| 17714 | CTCACAGTCAGCATACTGAG | Reverse 8' |  |
| 17609 | CTACACGTAAGATGCCACACC | Sens 8'' |  |
| 18895 | GTGTGCGACCATTTTTCCAGG | Reverse 8 |  |
