# Supplementary material for: Antiviral efficacy of favipiravir against Ebola virus: A translational study in cynomolgus macaques
Source: PLoS Med. 2018 Mar 27;15(3):e1002535. doi: 10.1371/journal.pmed.1002535 (PMC5870946; doi:10.1371/journal.pmed.1002535)
Supplement: S5 Table — The number of synonymous and non-synonymous variants was considered excluding the part of the GP gene that encodes several ORFs. Proportion of non-synonymous variants expected under a regimen of random distribution: 78%. (DOCX) [file pmed.1002535.s010.docx]

**S5 Table.** Proportion of non-synonymous variant sites over time in the different therapeutic groups. The number of synonymous and non-synonymous variants was considered excluding the part of the GP gene that encodes several ORFs. Proportion of non-synonymous variants expected under a regimen of random distribution: 78%.

|  |  | Treatment group | | |
| --- | --- | --- | --- | --- |
| Day post-infection | Untreated | 100 mg/kg BID | 150 mg/kg BID | 180 mg/kg BID |
| 0 | 73% | 73% | 73% | 73% |
| 5 | 53% | 59% | 62% | 61% |
| 7 | 48% | 59% | 56% | 55% |
| 8-10 | 54% | 51% | 53% | 51% |
