# Supplementary material for: Antiviral efficacy of favipiravir against Ebola virus: A translational study in cynomolgus macaques
Source: PLoS Med. 2018 Mar 27;15(3):e1002535. doi: 10.1371/journal.pmed.1002535 (PMC5870946; doi:10.1371/journal.pmed.1002535)
Supplement: S1 Text — (DOCX) [file pmed.1002535.s014.docx]

**Antiviral efficacy of favipiravir against Ebola virus: a translational study in cynomolgus macaques: supporting information**

Jérémie Guedj^1§*^, Géraldine Piorkowski^2§^, Frédéric Jacquot^3^, Vincent Madelain^1^, Thi Huyen Tram Nguyen^1^_,_ Anne Rodallec^2,4^, Stephan Gunther^5^, Caroline Carbonelle^3^, France Mentré^1§^, Hervé Raoul^3§^_,_ Xavier de Lamballerie^2§^

**Affiliations:**

^1^IAME, UMR 1137, INSERM, Université Paris Diderot, Sorbonne Paris Cité Paris, France ; ^2^UMR "Émergence des Pathologies Virales" (EPV: Aix-Marseille University - IRD 190 - Inserm 1207 - EHESP), Marseille, France; ^3^Laboratoire P4 Inserm-Jean Mérieux, US003 Inserm, Lyon, France; ^4^SMARTc Unit, U911 Cro2 Aix-Marseille University, Marseille, France; ^5^Bernhard Nocht Institute for Tropical Medicine, Hamburg, Germany.

**Running title:** Efficacy of favipiravir against Ebola in non-human primates

^§^: equally contributed

* Address correspondence to [jeremie.guedj@inserm.fr](mailto:jeremie.guedj@inserm.fr)

# S1 text: Determination of high dose of favipiravir using a viral kinetic model

Here we explain how the choice for the doses of 150 and 180 mg/kg BID was made based on the analysis of viral load data collected in untreated NHPs and in NHPs treated with 100 mg/kg BID.

# Methods

## Data

Viral load data in 20 NHPs were obtained from two experiments:

- Study A. Eight Mauritian female cynomolgus infected with 10 or 1000 ffu of EBOV Gabon virus (4 monkeys/group) and not treated from a previously published study [1].
- Study B. Twelve Mauritian female cynomolgus not treated (N=6). This includes the 6 NHPs from experiment 1 (Figure 1A) and infected with 1000 ffu (experiment 1 N=3 treated ; N=3 untreated) and 6 NHPs that received the same protocol but were infected with 10 ffu (N=3 treated ; N=3 untreated)

## Viral kinetic model

As long as target cells are not depleted, the growth of the virus can be explained using the simple model([*35*](#_ENREF_35)):

$${VL}_{t}={VL}_{0} exp(\delta{(R}_{0}-1)t)$$

Where VL_t_ (resp. VL_0_) is the viral load at time *t* (resp. time=0), R_0_ is the basic reproduction number, i.e., the number of cells that can be infected by a single infected cell during its lifespan, and δ is the loss rate of infected cells. Because only the product of R_0_ and δ can be estimated, we fixed δ to 1 without loss of generality and estimated only VL_0_ and R_0_. To take into account the difference in inoculum size, we assumed that the VL_0_ obtained with 10 ffu was 100 times lower than that that obtained with 1000 ffu.

The model was used to fit viral load data until day 7. The effect of the treatment, noted ε, was modeled using an E_max_ model where ε=C/(C+C_50_) and C_50_ is the concentration leading to 50% reduction in R_0_ and C is the average concentrations. C was equal 0 in untreated NHPs and was assumed similar in all treated NHPs and equal to 62.2 mg/L, the median predicted value at day 7 using a pharmacokinetic model of favipiravir in cynomolgus [2]. The effect of inoculum size and treatments were tested on R_0_.

## Predictions for viral load kinetics with doses of 150 and 180 mg/kg BID

Using the parameter estimated, 1,000 *in silico* animals were simulated assuming the same distribution in viral kinetic parameters as observed in studies A and B. In order to predict the effect of increasing dose of favpiravir and following the same approach than during the estimation step, we assumed conservatively that drug concentrations were equal to the average concentrations predicted by our model with 150 or 180 mg/kg BID [2].

# Results

## Data fitting

Animals treated with 100 mg/kg BID had significantly lower viral loads that untreated, both at days 5 and 7 (P=0.04 and 0.009, respectively, Table S1). The median time to death was equal to 9 and 10.5 days in untreated vs treated NHPs, respectively, but this difference was not significant (P=0.16). The model parameter estimates are given in Table S2.

## Model predictions

The model predicted that 150 mg/kg BID would lead to a median reduction of 3.5 and 2.8 log_10_ RNA/mL at day 7 with 10 or 1,000 ffu compared to untreated NHPs, respectively. The model predicted that 180 mg/kg would lead to a median reduction of 5 and 4 log_10_ RNA/mL at day 7 with 10 or 1000 log_10_ RNA/mL compared to untreated NHPs, respectively (Table S3). Interestingly when the experiments 2 and 3 were performed (see Table 1) we found that results were in close agreement with our predictions, with median values at D5 of 4.13 and 3.56 at 150 and 180 mg/kg BID (Table S3)

S1 Text Table1. EBOV RNA (log_10_ copies/mL) in untreated and treated NHPs

| Viral load at | **10 ffu** | | **p-**  **value** | **1000 ffu** | | **p-**  **value** | **10+1000 ffu** | | **p-**  **value** |
| --- | --- | --- | --- | --- | --- | --- | --- | --- | --- |
|  | **untreated** | **treated** |  | **untreated** | **treated** |  | **untreated** | **treated** |  |
| day 5 | 5.88 | 4.96 | 0.07 | 6.46 | 6.17 | 0.38 | 6.17 | 5.06 | 0.04 |
| day 7 | 9.09 | 7.55 | 0.07 | 8.92 | 6.86 | 0.067 | 9.0 | 7.29 | 0.009 |

S2 text Table 2. Parameter estimates of the viral kinetic model

| Parameters | Estimate value (Standard error) |
| --- | --- |
| VL_0_ (log_10_ copies/mL) | -0.74 (0.45) |
| R_0_ (Inoculum 10 ffu) | 4.08 (0.19) |
| R_0_ (Inoculum 1000 ffu) | 3.36 (0.19) |
| C_50_ (µg/mL) | 565 (190) |

S3 Text Table3. Model median predictions (min-max) for the viral load (log_10_ RNA/mL) at days 5 and 7 according to the dose of favipiravir and the inoculum size administered

|  | **10 ffu** | | **1000 ffu** | |
| --- | --- | --- | --- | --- |
|  | **Day 5** | **Day 7** | **Day 5** | **Day 7** |
| **150 mg/kg** | 3.74 (3.48-4.09) | 5.53 (5.17-6.02) | 4.57 (4.36-4.85) | 5.89 (5.59-6.29) |
| **180 mg/kg** | 2.45 (1.29-3.13) | 3.73 (2.10-4.68) | 3.51 (2.54-4.07) | 4.40 (3.06-5.19) |
|  | Results observed in Experiments 2 and 3 | | | |
| **150 mg/kg** | NA | NA | 4.13 | 5.94 |
| **180 mg/kg** | NA | NA | 3.56 | 4.38 |

**References**

1. Piorkowski G, Jacquot F, Quérat G, Carbonnelle C, Pannetier D, Mentré F, et al. Implementation of a non-human primate model of Ebola disease: Infection of Mauritian cynomolgus macaques and analysis of virus populations. Antiviral Res. 2017;140: 95–105.

2. Nguyen THT, Guedj J, Anglaret X, Laouénan C, Madelain V, Taburet A-M, et al. Favipiravir pharmacokinetics in Ebola-Infected patients of the JIKI trial reveals concentrations lower than targeted. PLoS Negl Trop Dis. 2017;11: e0005389.
