# Supplementary material for: Antiviral efficacy of favipiravir against Ebola virus: A translational study in cynomolgus macaques
Source: PLoS Med. 2018 Mar 27;15(3):e1002535. doi: 10.1371/journal.pmed.1002535 (PMC5870946; doi:10.1371/journal.pmed.1002535)
Supplement: S2 Text — (DOCX) [file pmed.1002535.s015.docx]

**Antiviral efficacy of favipiravir against Ebola virus: a translational study in cynomolgus macaques: supporting information**

Jérémie Guedj^1§*^, Géraldine Piorkowski^2§^, Frédéric Jacquot^3^, Vincent Madelain^1^, Thi Huyen Tram Nguyen^1^_,_ Anne Rodallec^2,4^, Stephan Gunther^5^, Caroline Carbonelle^3^, France Mentré^1§^, Hervé Raoul^3§^_,_ Xavier de Lamballerie^2§^

**Affiliations:**

^1^IAME, UMR 1137, INSERM, Université Paris Diderot, Sorbonne Paris Cité Paris, France ; ^2^UMR "Émergence des Pathologies Virales" (EPV: Aix-Marseille University - IRD 190 - Inserm 1207 - EHESP), Marseille, France; ^3^Laboratoire P4 Inserm-Jean Mérieux, US003 Inserm, Lyon, France; ^4^SMARTc Unit, U911 Cro2 Aix-Marseille University, Marseille, France; ^5^Bernhard Nocht Institute for Tropical Medicine, Hamburg, Germany.

**Running title:** Efficacy of favipiravir against Ebola in non-human primates

^§^: equally contributed

* Address correspondence to [jeremie.guedj@inserm.fr](mailto:jeremie.guedj@inserm.fr)

# S2 text: drug concentrations in infected and uninfected NHPs

## Data

The following data were used:

- N=9 animals receiving 200 mg/kg BID as loading dose and 100 mg/kg BID as maintenance dose. This includes the 3 treated NHPs from experiment 1 (Figure 1A), 3 NHPs that received the same protocol but were infected with 10 ffu and 3 NHPs that received the same protocol but were not infected
- N=9 animals receiving 250 mg/kg BID as loading dose and 150 mg/kg BID as maintenance dose. This includes the 5 treated NHPs from experiment 2 (Figure 1A) and 4 NHPs that received the same protocol but were not infected
- N=10 animals receiving 250 mg/kg BID as loading dose and 180 mg/kg BID as maintenance dose. This includes the 5 treated NHPs from experiment 3 and 5 NHPs that received the same dosing regimen for 7 days but were not infected. These NHPs were not handled in the BSL4, but received the same protocol (twice daily injection and anesthesia) and were part of a previous tolerance and PK study [1].

Uninfected animals were euthanized at day 13 and day 21 in the 100 and 150 mg/kg BID groups, respectively.

### Methods

Trough concentrations at days 2 (when available), 5 and 7 post treatment initiation (that correspond do days 4, 7 and 9 post infection in infected animals, respectively) were compared across dosing groups. Comparison between groups at each time point was assessed using a two sided Wilcoxon test and a P-value of 0.05 was considered significant.

### Results

There was no statistically significant differences at any of the time point considered (all P-values >0.50). We display in Figure S1 the median drug concentrations observed across dosing groups in infected and uninfected animals. We overlaid for the same of the comparison the comparison observed in patients with EVD from the JIKI trial [2]. We also display in Figure S2 the weight, temperatures, hemoglobin and creatinine levels observed in infected and uninfected animals receiving favipiravir, parameters that could be affected by treatment even in absence of infection [1].


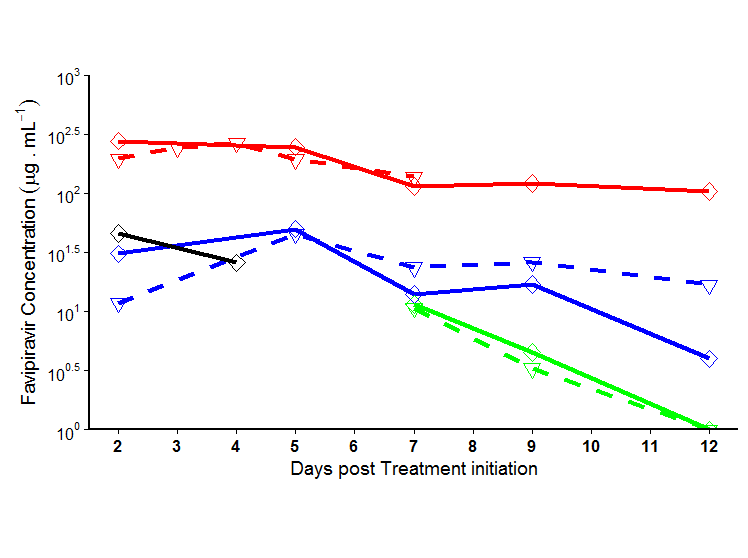


**S2 Text Fig 1.** Median trough concentrations in infected and uninfected animals. Dotted lines indicate non-infected animals and solid line indicate infected animals (infected 2 days after treatment initiation). For NHPs treated with 180 mg/kg BID, there was no treated uninfected animals and we displayed animals treated with the same protocol in a previous study([*13*](#_ENREF_13)). Green: 100 mg/kg BID; blue: 150 mg/kg BID; red: 180 mg/kg BID. The black line are the trough concentrations found in patients with EVD in the JIKI trial [2]. NB: median data may be calculated on different number of animals overtime, depending on data availability and survival.


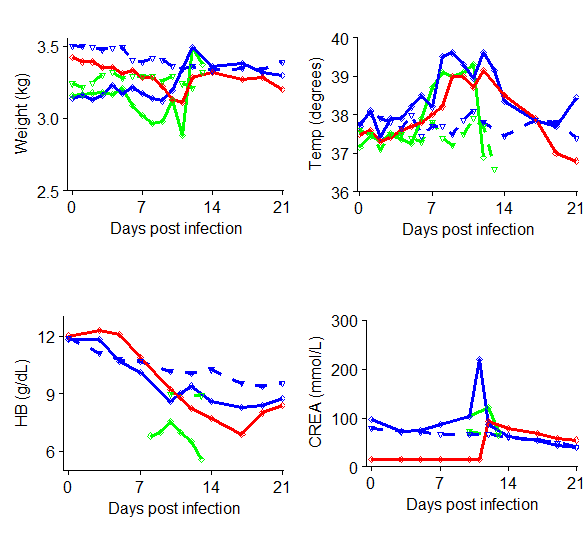


**S2 Text Fig 2**. Median evolution of Temperature (Temp), Weight (Weight), Haemoglobin (HB) and Creatinine (CREAT) in infected or uninfected animals receiving favipiravir. Dotted lines indicate non-infected animals and solid line indicate infected animals. Green: 100 mg/kg BID; blue: 150 mg/kg BID; red: 180 mg/kg BID. NB: median data may be calculated on different number of animals overtime, depending on data availability and survival.

**References**

1. Madelain V, Guedj J, Mentré F, Nguyen THT, Jacquot F, Oestereich L, et al. Favipiravir Pharmacokinetics in Nonhuman Primates and Insights for Future Efficacy Studies of Hemorrhagic Fever Viruses. Antimicrob Agents Chemother. 2017;61: e01305-16.

2. Nguyen THT, Guedj J, Anglaret X, Laouénan C, Madelain V, Taburet A-M, et al. Favipiravir pharmacokinetics in Ebola-Infected patients of the JIKI trial reveals concentrations lower than targeted. PLoS Negl Trop Dis. 2017;11: e0005389.
