# Supplementary material for: Antiviral efficacy of favipiravir against Ebola virus: A translational study in cynomolgus macaques
Source: PLoS Med. 2018 Mar 27;15(3):e1002535. doi: 10.1371/journal.pmed.1002535 (PMC5870946; doi:10.1371/journal.pmed.1002535)
Supplement: S3 Text — (DOCX) [file pmed.1002535.s016.docx]

**Antiviral efficacy of favipiravir against Ebola virus: a translational study in cynomolgus macaques: supporting information**

Jérémie Guedj^1§*^, Géraldine Piorkowski^2§^, Frédéric Jacquot^3^, Vincent Madelain^1^, Thi Huyen Tram Nguyen^1^_,_ Anne Rodallec^2,4^, Stephan Gunther^5^, Caroline Carbonelle^3^, France Mentré^1§^, Hervé Raoul^3§^_,_ Xavier de Lamballerie^2§^

**Affiliations:**

^1^IAME, UMR 1137, INSERM, Université Paris Diderot, Sorbonne Paris Cité Paris, France ; ^2^UMR "Émergence des Pathologies Virales" (EPV: Aix-Marseille University - IRD 190 - Inserm 1207 - EHESP), Marseille, France; ^3^Laboratoire P4 Inserm-Jean Mérieux, US003 Inserm, Lyon, France; ^4^SMARTc Unit, U911 Cro2 Aix-Marseille University, Marseille, France; ^5^Bernhard Nocht Institute for Tropical Medicine, Hamburg, Germany.

**Running title:** Efficacy of favipiravir against Ebola in non-human primates

^§^: equally contributed

* Address correspondence to [jeremie.guedj@inserm.fr](mailto:jeremie.guedj@inserm.fr)

# S3 text: Genomic analysis

Extraction protocol

Extraction of the serum was realised using Cador Pathogen kit (Qiagen) and was performed with the QIAcube HT device (Qiagen) following manufacturer’s instructions. Final elution was performed in 80µL of AVE buffer without top Elute fluid. The extraction was realised as described in [1].

*Clinical samples and inoculum RT-PCR*

Eight overlapping PCR amplicons were generated from 3 µL of viral RNA, 400nm of each primer (Table S4) and the SuperScript® III One-Step RT-PCR System with Platinum® *Taq* High Fidelity kit (ThermoFisher Scientific) (12.5µl of reaction mix and 0.5µl of RT/Taq mix) in 25µl final volume. The thermal profile used was 30 min at 50ºC, 5 min at 94ºC, 45 cycles of 15 sec at 94ºC, 30 sec at 56 ºC, 4 min at 68ºC, ending with 5 min at 68ºC. The amplicons were subsequently purified using the NucleoFast 96 PCR plate (Macherey-Nagel) and quantified using Nanodrop 1000 device (ThermoFisher Scientific) following manufacturer’s instructions.

*Next Generation Sequencing*

For each pool, the dsDNA concentration was quantified using Qubit® dsDNA HS Assay Kit and Qubit 2.0 fluorometer (ThermoFisher Scientific). After a physical fragmentation (sonication) in 200bp fragments, libraries were built using the automated AB Library Builder System (ThermoFisher Scientific). To pool equimolarly the barcoded samples a quantification step by the 2100 Bioanalyzer instrument (Agilent Technologies) was realised. The emulsion PCR of the pools and the loading of the 530 chips was realised using the automated Ion Chef instrument (ThermoFisher Scientific). The sequencing reaction was performed on the S5 device (ThermoFisher Scientific). A plasmidic control was realized as described in [1].

*Data analysis*

Automated read datasets provided by Torrent software suite 5.2.1 were trimmed according to quality score (99%) and length (reads shorter than 100bp were removed) using CLC genomics workbench software (CLC bio-Qiagen). Primers used for RT-PCR were removed using an in-house software package.

The consensus sequence, provided by CLC genomic software (majority base at each position) (Qiagen), was manually checked. Errors like insertions or deletions were manually corrected. To study variants, the 30 first and 30 last nucleotides of each reads were removed, and mapped on the consensus sequence (the read extremities have a lower quality than the nucleotides in the middle). Mutation frequencies of nucleotides at each position were calculated as the number of reads of the concerning nucleotide divided by the total number of validated reads at that site. A major variant was defined as a change in the sequence, relative to the reference sequence (frequency of mutations >50%). Substitutions with a frequency higher than 1% were selected for further analysis of minor variants.

*Distribution of variants at synonymous and non-synonymous sites*

The distribution of identified variants at synonymous and non-synonymous sites was compared with an in-house model providing random distribution of the same number of variant sites in EBOV coding regions after 1,000 simulation replicates (see [1] for analysis description).

**References**

1. Piorkowski G, Jacquot F, Quérat G, Carbonnelle C, Pannetier D, Mentré F, et al. Implementation of a non-human primate model of Ebola disease: Infection of Mauritian cynomolgus macaques and analysis of virus populations. Antiviral Res. 2017;140: 95–105.
