# Supplementary material for: Antiviral efficacy of favipiravir against Ebola virus: A translational study in cynomolgus macaques
Source: PLoS Med. 2018 Mar 27;15(3):e1002535. doi: 10.1371/journal.pmed.1002535 (PMC5870946; doi:10.1371/journal.pmed.1002535)
Supplement: S4 Text — (PDF) [file pmed.1002535.s017.pdf]

of European research in the international preparedness and response programme for the monitoring and control of human and veterinarian emerging infectious diseases such as Ebola.

The use of Erinha, EVA, SILVER and EMLab capacities represents a main advantage for a rapid implementation of the proposed scientific programme as well as for optimising research outcomes by pooling needed scientific information, expertise and infrastructures.

The project is conceived as a three-step, scalable and evolving programme as described hereafter.

Task 3.1: favipiravir PK & tolerance studies.
Coordinator: X de Lamballerie (AMU)

Subtask 3.1.1 will include the analysis of information provided by the Favipiravir manufacturer for humans and NHPs. Results from PK experiments performed in the same NHP model by Toyama (the manufacturer of the drug) -ongoing at the time of redaction of the current proposal- will be made available to the consortium shortly and will provide pivotal information for the design of experiments. Toyama's experiments are performed using both the oral and IV routes. The analysis of the PK data in NHPs will be used to build a model based approach for drug PK in non-infected NHPs. The model will be analysed with non-linear mixed effect models, the recommended approach in PK modelling which borrow strength from the whole sample to more precisely estimate the population parameters of the model, such as the mean PK parameters (Volume of distribution, clearance) and the inter-individual variation (IIV). Parameter will be estimated by maximum likelihood approach and the software MONOLIX. Different type of models will be introduced and will be compared Bayesian Information Criterion (BIC), a criterion used to compare the quality of the fits of different models, accounting for the number of parameters (the lower the better).
The target concentration to be used in this NHP study is based on previous studies performed in mice in which the drug exposure was established in uninfected mice that received the therapeutic dose (for EBOV) of 300 mg/kg (Oestereich et al., 2014). Using these data, several candidate doses will be proposed for NHP, corresponding to exposures equal to 10%, 50% and 100% of the target concentrations found in these previous studies.
Importantly, because the development of a challenging infected NHP model, we will optimize the times for the sampling measurements in order to obtain precise PK parameters in spite of the limited number of sampling measurements. This will be done using PFIM, a software developed by the group of France Mentré for optimizing the information collected during PK studies (see aslo WP4).

Subtask 3.1.2 will include de novo experiments that will be scaled according to the information made available in the above mentioned analysis steps. It will take advantage of the available macaque model implemented in the Lyon-Mérieux BSL4 lab (see Task 3.2 for more details) which is similar to the model used by Toyama. Experiments in non-infected animals will be subcontracted in Strasbourg (Silabe, Stockbreeder of animals). A specific Animal facilities level 2 will be dedicated for this experiment. Injection of Favipiravir and blood samples will be performed by veterinarian staff under the supervision of the consortium. Favipiravir dosage will be subcontracted to a specific partner of Silabe (laboratory able to measure Favipiravir in blood sample, identification of laboratory in progress). Dosage procedures will include the evaluation of the impact of inactivation protocols to determine whether PK studies can be extended to infected animals (see Task 3.3)

Importantly, the management of monkeys will imply iterative anaesthesias for each medication. Since the half-life of favipiravir is short, the usual treatment regimen is twice a day (BID). Consequently, the favipiravir PK and tolerance studies will include standard BID oral and IV experiments, but will also decipher the possibility to mimic maintenance treatment using alternative methods. This may include the use of permanent IV infusion systems allowing to reduce the duration of anaesthesias and to facilitate blood sampling. The analysis of Favipiravir kinetics in plasma will indicate which method is the most relevant for use in infected animals.
Accordingly, NHP experiments will be performed using groups of 3 Cynomolgus macaques. Loading and maintenance doses as well as medication routes will be tested, starting from the experimental and modelling information provided by the manufacturer, and aiming at characterising how plasma concentrations in the range of expected antiviral efficacy can be reached in a short delay and maintained during the treatment.
Biological sampling will allow dosage of the drug and follow-up of clinical biochemical and haematology parameters.

The final objective will be to choose the optimal favipiravir dose(s), time and route for medication used in subsequent infection experiments and work out an experimental protocol for Task 3.2. This includes gaining new information about the loading dose necessary for obtaining as rapidly as possible plasma concentrations in the range where antiviral efficacy is expected, and about the maintenance dose that would allow to sustain efficient treatment. From the combination of previous human PK studies and experiments in mouse models it can be evaluated that maintaining

plasma concentrations above ca. 60 µg/mL is required and that this range of concentration can be obtained using both the oral and IV routes.

Task 3.2: NHP EBOV infections & Favipiravir treatment.
Coordinator: H Raoul (P4LM)

Subtask 3.2.1 will aim at designing from Task 3.1 results the best experimental protocol to test the antiviral activity of favipiravir in monkeys infected by EBOV.

Macaque model
The macaque model implemented in the Lyon-Mérieux BSL4 lab is a lethal model relying on the IM injection of 100 ffu of Zaire EBOV in male macaques. This is a "severe" model compared with natural infection in humans, that quite perfectly mimics accidental infection (e.g., by an infected needle or following a laboratory accident). An important consequence is the short duration of the incubation period, with macaques becoming febrile at day 4 and dying around day 7.

Doses and routes of administration
Testing different doses dramatically increases the number of NHPs necessary for experiments. Therefore, the number of doses tested will be reduced to the minimum by combining various information issuing from Task 3.1 (experimental PK studies, tolerance, data from the manufacturer). As it seems probable that quite high doses may be necessary, the highest well-tolerated doses will be tested.

As reported in the previous sections, any route of administration will be possible in the pre-exposure period and also for giving the loading dose in the post-exposure period. The maintenance treatment in infected animals will be performed according to the most relevant method identified in Task 3.1.

Prophylactic administration will be performed by the oral route exclusively and will investigate the possibility to prevent infection and/or disease by providing to exposed animals a pre-exposure oral treatment that will be pursued for 10 days post infection (pi) using the most appropriate maintenance route for medication. Different doses will be tested to determine which plasma concentration of the drug is necessary to provide protection.

Post-exposure treatments will be started using the IV route and pursued using the most appropriate maintenance route for medication. Treatment protocols will be designed for a standard duration of 10 days and increasing doses will be tested to estimate the conditions (time from infection, Favipiravir doses) in which antiviral efficacy can be observed along the natural history of infection.

Experimental strategy
The ideal design would rely on estimating the protective effect of favipiravir at an early stage of the disease and, if such protection can be obtained, proceeding to experiments at a later stage of the disease, otherwise repeating the experiment in different conditions (e.g., with increased doses). This is summarised in the diagram hereafter where each treatment step is performed only if the previous one was successful.

However, such a strategy would imply occupying the BSL4 laboratory for an extremely long period with a limited number of animals, since each experimental series will last for at least 4 weeks (1 week for acclimation of newly arrived animals and 3 weeks for infection and treatment follow-up). Therefore, the final design will combine this global go/no go strategy with an optimisation of the use of the animal facility, in which 15 monkeys can be studied simultaneously. It is estimated that a complete study protocol will require 4 series of 4-week experiments, starting with one series dedicated to control (infection without treatment) and pre-exposure experiments, followed by three series dedicated to favipiravir time-of addition post-exposure experiments along the asymptomatic and symptomatic phases of EBOV infection.

Subtask 3.2.2 will be dedicated to NHP management & experiments and adaptive follow-up.
Experiments will be performed in the Lyon-Mérieux BSL4 laboratory, following the above mentioned strategy and using groups of 3 Cynomolgus macaques equipped with IPTT300 transponders or Anipill sensors. Survivors will be euthanized at day 21 by injection of Dolethal® (intracardiac route) and all monkeys will be autopsied (including anatomopathological analysis when necessary).

Groups will be designed on the basis of criteria that include: PK & tolerance analyses, route of administration, medication schedule, EBOV infection, with special attention to the impact of antiviral treatment started in the pre-exposure period and at the early stages of the post-exposure period.

Clinical and biological follow-up will be organised as described hereafter, with daily blood sampling (up to 5 mL per day).

Task 3.3: Data collection and analysis.
Coordinator: S Günther (BNI)

Subtask 3.3.1 will be dedicated to clinical, biochemistry and haematology data.
The clinical follow-up will be performed in a standardised manner by the BSL4 laboratory team and will include:
- Daily weighing and temperature recording during the treatment and three times a week without treatment
- Report of clinical presentation and activity
- Blood sampling

Biochemistry and haematology tests will all be performed within the BSL4 laboratory and will include (i.a.):
- Blood cell count
- Electrolytes
- hepatic enzymes
- uric acid

The monitoring and final assessment will be performed by the Porton Down team to allow a comprehensive comparative analysis between the different groups of monkeys.

Subtask 3.3.2 will be dedicated to virus molecular detection and measurement of viral load.
A standardised aliquot of serum will be inactivated by the addition of AVL buffer according to a validated procedure. This inactivated solution will be submitted to extraction of nucleic acids that will be sent to BNI for performing qualitative detection of EBOV genome and molecular measurement of viral load using established real-time RT-PCR techniques. The latter will be performed using quantified synthetic RNA controls.

Subtask 3.3.3 will be dedicated to virus evolution and NGS analyses. Genome characterisation will be performed directly from blood nucleic acids extract using Next Generation Sequencing (NGS). Briefly, long overlapping PCR fragments spanning the entire genome will be prepared using EBOV Zaire-specific primers and a high fidelity amplification kit. The PCR products will be purified and 200 ng used for characterisation with the Ion PGM Sequencer (Rothberg et al., 2011) according to the manufacturer's instructions. The reads obtained will be treated using previously established trimming and quality score proprietary protocols, and further used to reconstruct de novo contigs. Analyses will address evolutionary issues for both fixed mutations and minority viral populations taking into account the clinical history and the course of virology infection. Specific attention will be paid to non-synonymous mutations occurring in the polymerase gene, since the main antiviral mechanism of favipiravir is expected to be the inhibition of the virus RNA-dependent RNA polymerase.

In addition, even though the hypothetical emergence of resistant-mutants in humans based on the treatment with Favipiravir is expected to be an insignificant epidemiological issue, the REACTION programme will analyze the evolution of viral sub-populations in NHPs receiving Favipiravir treatment. Whether emergence of mutants in the polymerase gene would be observed, this would be a strong argument for implementing combination therapy.

Subtask 3.3.4 will be dedicated to serological analyses. The humoral response induced during infection will be monitored using ELISA and seroneutralisation tests. Briefly, Ebola virus-specific IgM and IgG will be detected using an IgM- or IgG-capture ELISA and the presence of EBOV neutralising antibodies will be evaluated thanks to a seroneutralisation assay onto Vero E6 cells. The kinetic of humoral response will be followed throughout the course of the disease and until necropsy of surviving animals. For detection of specific IgA, an IgA-capture ELISA will be set up as this assay is not yet available but reagents for detection in macaques are commercially available. All experiments will be performed in a BSL4 environment.

Subtask 3.3.5 will be dedicated to other immunological studies, taking advantage of the availability of a large range of commercial assays analyses.

Subtask 3.3.5 will be dedicated to other immunological studies with a focus on T cell and dendritic cell (DC) function. These analysis will follow up on recent data generated by the consortium partners, which indicate that infection of DCs and upregulation of T cell inhibitory molecules such as CTLA-4 in humans is positively correlated with poor immunity and death (manuscript in preparation). We have designed NHP-specific 8- parameter flow cytometry panels to evaluate the T cell immunophenotype in peripheral blood over the course of favipiravir treatment, doable in the Hamburg BSL4 lab where a 10-color flow cytometer is available. These immunophenotipic studies will be completed with serum analysis of cytokines using Luminex technology, intracellular cytokine staining to assess the capacity of T

cells to respond to EBOV antigens (peptide pools or recombinant GP), and T-cell proliferation assays using cell division markers such as CFSE. To assess DC function we have similarly designed a 9-color panel to determine the functional status of peripheral blood DC subsets (expression of T cell co-stimulatory molecules) as well as their infection status (surface staining of EBOV GP). All experiments will be done in Lyon, BNI and DH BSL4 laboratories.

Subtask 3.3.6 will be dedicated to PK and bio-statistical analysis.
The analysis of favipiravir PK in infected animals would be of utmost interest but requires a standardised inactivation protocol (e.g., 60°C, 1 hour). Such a protocol will be evaluated in Task 3.1. If favipiravir dosage is impossible from inactivated sera, the antiviral activity of sera may be estimated within the P4 laboratory using a short inactivation protocol and cell culture infected with seasonal influenza virus (this is made possible by the fact that favipiravir is a potent inhibitor of influenza viruses).

For bio-statistical analysis, the principal evaluation criterion will be survival.
PK parameters (AUC calculated by the trapezoidal method, Cmin, tmax, clearance) will be collected using non-compartment analysis.
The main efficacy criterion will be survival, and differences according to doses and delayed treatment will be analysed using non-parametric log-rank tests. Then the effect of several factors including PK parameters (doses, pre-dose concentration), viraemia kinetic parameters (zenith, slope of decline after treatment initiation, etc) and genetic diversity will be evaluated using multivariate semi-parametric Cox models.
Secondary analysis will include the comparison of viraemia, PK, clinical biochemistry and haematology, anatomopathological and immunology studies at different time points and across dosing groups. These markers will be described using arithmetic or geometric mean and comparison curves between the different dosing groups regimen will be performed using non-parametric Wilcoxon test. The comparison between the plasma drug exposure and the viraemia will be performed using Spearman coefficient in each dosing group. More refined analysis using physiopathological models will be developed subsequently (see WP4 translational approach).

TASK 3.4: Contingency plans and protocol optimization
During the course of the project, the consortium will continuously evaluate the project progress and optimize the protocols where necessary.

Possible adjustments may include:
1. Possible availability of an intravenous Favipiravir product that may allow the optimized administration;
2. If technically feasible, priority will be given to intravenous use of favipiravir using remote programmable pumps (to avoid anaesthesia twice a day); if not, the IV route may be used with short anaesthesia protocols; if not, the oral route will be used and PK studies using the same anaesthesia protocols will be performed to optimise the experimental design of treatment;
3. The programme is organised with a series of "go/no go steps". For example, we will shift to tests of post-exposure protection only if pre-exposure has been validated. If a step is not validated, it will be repeated with a modified, optimised protocol (please refer to the corresponding WP information);
4. If antiviral activity of the molecule could not be evidenced (extremely low probability according to available information), the study platform could be used to challenge the efficacy of other molecules (e.g., Brincidofovir) or treatments (e.g., polyclonal antibodies) alone or in combination therapy;
5. If effect sizes are different than expected, the consortium will consider adjusting the number of NHP to be used in the study;
6. Sequence virus recovered from treated NHPs to illustrate the lack of or presence of mutations in the gene encoding the drug target.

Participation per Partner

**Partner number and short name**	**WP3 effort**
1 - INSTITUT NATIONAL DE LA SANTE ET DE LA RECHERCHE MEDICALE (INSERM)	16.80
3 - BNI	6.00
4 - IP	5.40
6 - AMU	7.20
