# Supplementary material for: Antiviral efficacy of favipiravir against Ebola virus: A translational study in cynomolgus macaques
Source: PLoS Med. 2018 Mar 27;15(3):e1002535. doi: 10.1371/journal.pmed.1002535 (PMC5870946; doi:10.1371/journal.pmed.1002535)
Supplement: S6 Text — (DOCX) [file pmed.1002535.s019.docx]

Data file description

ID : animal ID

Time: sampling time

Y: marker measured

CENS: 0 for value above the limit of detection, 1 otherwise

GROUP: dose received (maintenance)

Study: experiment (P4-1 for study 1, P4-2 for study 2, P4-3 for study 3)

Y: marker value

Additional names

S6 Table

TYPE: marker measured (Type = 1 is molecular viremia, Type =2 are titers)

S7 Table

AMT: drug amount received

RATE: injection rate

PEAK: indicates whether the measurement corresponds to a peak or pre-dose measurement (PEAK = 1 if peak, 0 if this is a predose)
